# Subnanometer Topological Tuning of the Liquid Intrusion/Extrusion Characteristics of Hydrophobic Micropores

**DOI:** 10.1021/acs.nanolett.1c02140

**Published:** 2022-03-08

**Authors:** Yuriy G. Bushuev, Yaroslav Grosu, Mirosław A. Chora̧żewski, Simone Meloni

**Affiliations:** a Institute of Chemistry, University of Silesia in Katowice, Szkolna 9 Street, 40-006 Katowice, Poland; b Centre for Cooperative Research on Alternative Energies (CIC energiGUNE), Basque Research and Technology Alliance (BRTA), Alava Technology Park, Albert Einstein 48, 01510 Vitoria-Gasteiz, Spain; c Dipartimento di Scienze Chimiche, Farmaceutiche ed Agrarie (DOCPAS), Università degli Studi di Ferrara (Unife), Via Luigi Borsari 46, I-44121 Ferrara, Italy

**Keywords:** nanoporous materials, hydrophobic nanoparticles, solid−liquid interface, intrusion/extrusion

## Abstract

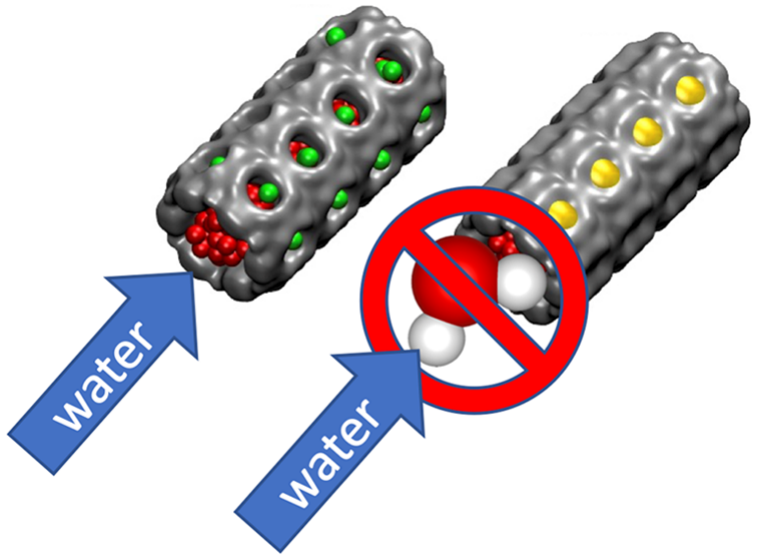

Intrusion (wetting)/extrusion (drying) of liquids in/from lyophobic nanoporous systems is key in many fields, including chromatography, nanofluidics, biology, and energy materials. Here we demonstrate that secondary topological features decorating main channels of porous systems dramatically affect the intrusion/extrusion cycle. These secondary features, allowing an unexpected bridging with liquid in the surrounding domains, stabilize the water stream intruding a micropore. This reduces the intrusion/extrusion barrier and the corresponding pressures without altering other properties of the system. Tuning the intrusion/extrusion pressures via subnanometric topological features represents a yet unexplored strategy for designing hydrophobic micropores. Though energy is not the only field of application, here we show that the proposed tuning approach may bring 20–75 MPa of intrusion/extrusion pressure increase, expanding the applicability of hydrophobic microporous materials.

Porous crystals, such as zeolites, metal–organic frameworks (MOFs), and covalent organic frameworks (COFs), are materials with many different applications such as catalysis, liquid/liquid and liquid/gas separation, chromatography, energy storage, and many more.^1−4^ Some of these applications are based on the (selective) intrusion/extrusion of liquids in/from these porous systems. The key to these applications is controlling the pressure at which liquids intrude (wet)/extrude (dry) a porous material. Increasing the intrusion pressure, for example, results in an increase of the energy stored during the process, bringing to an increase in the energy density of the material.^5,6^ On the contrary, reducing the intrusion pressure typically broadens the applications’ range and lowers the cost of using these materials for, e.g., liquids’ separation.^7^ Porous materials can also be used for damping vibrations or absorbing crashes: materials presenting sizable pressure hysteresis in the intrusion/extrusion cycle, that is, those materials in which the extrusion pressure is much lower than the intrusion one, transform the mechanical energy comprised within the cycle into some other form of energy, for example, thermal and/or electrical energy.^8^ In this case, tuning intrusion/extrusion pressures is useful to make the material characteristics consistent with operative conditions without using any pressure multiplier/demultiplier, thus reducing the complexity of energy damping devices.

Understanding the characteristics controlling the intrusion/extrusion of a pore, in particular, subnanometric features not considered so far, might have much broader implications. For example, one of the mechanisms of ion gating in biological systems, hydrophobic gating, depends on the (tunable) wettability of hydrophobic pores,^9−11^ which can also be dynamically driven by external stimuli. Our findings might inspire design principles for switchable bionanopores.

To tune intrusion/extrusion characteristics, one typically acts on the chemical nature of the system, for example, on the type of ligands used to synthesize MOFs, and on the size of the main pores where liquid intrusion occurs.^12^ Previous works also considered the introduction and tailoring of a hierarchy of pores in zeolites, namely, the creation of the second level of larger pores, mesopores, to enhance the accessibility of the interior of crystalline grains^13^ or to augment the space available to the liquid, *V*_*pores*_, to increase the energy that can be stored in the material.^14^ Here, on the contrary, we focus on even smaller pores, the secondary subnanometer pores decorating the main cavities of zeolites, MOFs and COFs, that can be present in the original framework or synthesized on purpose to tune their intrusion/extrusion characteristics. We perform atomistic simulations to show that these secondary subnanometric porosities can be exploited to largely extend the tunability and control of the intrusion/extrusion pressure without impairing other functional properties of the system, such as intrusion/extrusion hysteresis and the main chemistry of the system. We remark that the objective of this work is to identify novel design principles that, in conjunction with traditional strategies based on altering the chemistry and geometry of the main channels, can inspire strategies to fabricate materials with tailored intrusion/extrusion characteristics. Here we do not focus on synthetic rules to implement these strategies in specific classes of materials or biological systems. However, to provide an example, one can imagine tuning subnanometric apertures of a specific subfamily of the zeolitic imidazolate framework (ZIF) MOF by modifying the residue attached to the C2 carbon, which affects some/all apertures of the porous material.^15^

To illustrate this principle, we first focus on a putative pure silica zeolite of ITT-type framework^16^ (Figure 1), named after the synthetic ITQ-33 (Thirty-Three) zeolite.^100^ Our computer experiments shows to have a shock-absorber-like behavior, that is, a pronounced intrusion/extrusion pressure hysteresis (Figure 2a). Then, we also considered the case of silicalite-1 (Figure 2b), a zeolite of MFI-type framework with molecular spring behavior (small/negligible hysteresis).^100,17^ The MFI framework in named after the ZFM-FIve zeolite (Zeolite Socony Mobil with sequantial number five). Validation of the general principles identified in this work based on only two, though significantly different, zeolites is insufficient to prove the generality of the subnanometer topological tuning principle, and more work will be necessary in the future; nevertheless, it is reassuring that the phenomenon is not specific to one system and its peculiar characteristics.

**Figure 1:**
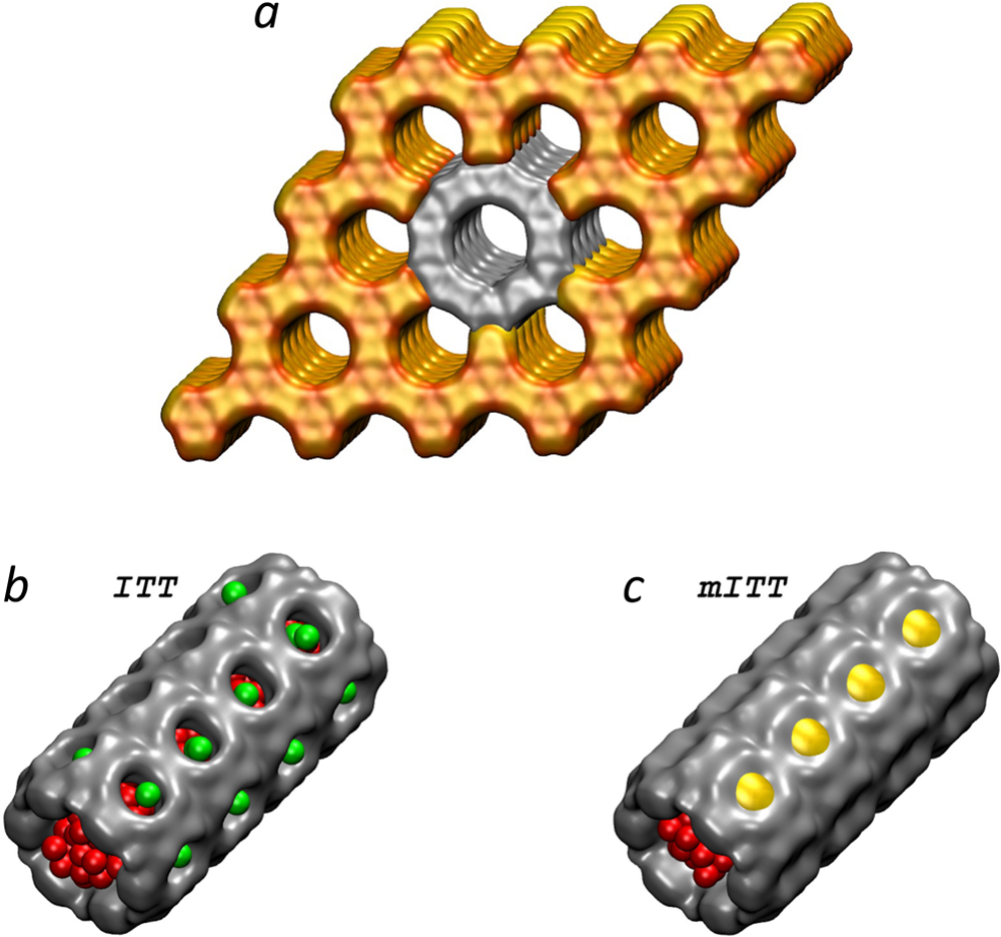
(*a*) Crystalline grain of ITT-type zeolite used for simulations. The sample is shown considering the volume precluded to water due to the steric hindrance of atoms of the framework. Eighteen MR channels of ITT (*b*) and mITT (*c*) filled by water; red and green spheres depend on whether they lie in 18 MR channels or 10 MR windows, respectively. For mITT, the 10 MR windows are closed by -Si–O–Si-bridges (yellow shutters).

**Figure 2:**
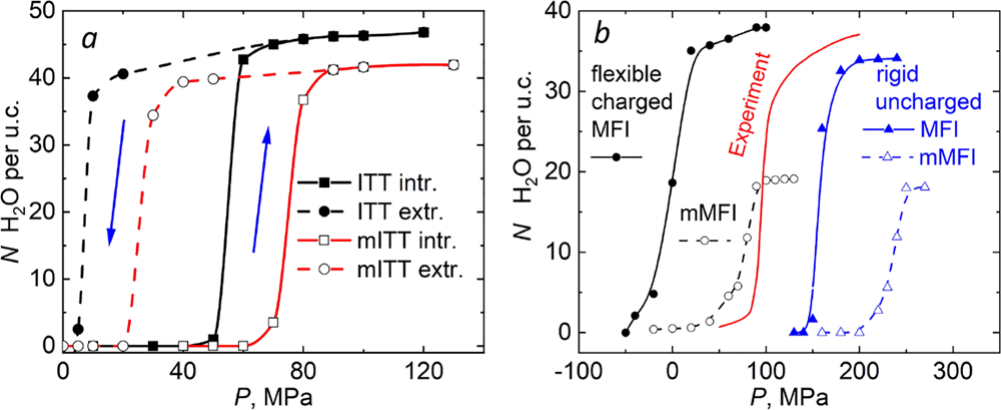
(*a*) Intrusion/extrusion isotherms for ITT/mITT calculated for rigid/uncharged models. (*b*) Intrusion isotherms for MFI/mMFI-type zeolites calculated for rigid/uncharged and flexible/charged models.

The pure silica ITT contains hexagonal channels running along the [001] axis consisting of 18 silicon and 18 oxygen atoms rings, 18-membered ring channels—18MR (Figure 1a), with a pore aperture of 1.53 nm. Each channel features 10MR lateral windows with an aperture of 0.78 nm (Figure 1b). 10MR windows put 18MR cavities in contact with surrounding channels and bulk water if they lie on the surface of a crystallite (Figure 1a). To test the effect of these 10MR lateral pores, we considered a second, *artificial*, zeolite with a modified ITT framework (mITT), obtained by bridging oxygen atoms laying at the opposite sides of 10MR windows by -Si–O–Si-chains (yellow shutters in Figure 1c). This bridging turns the interconnected 3D network of pores of ITT into a system of isolated 1D channels. This is possible thanks to the flexibility of computer simulations, which allow one to create a putative zeolite violating the coordination of bridged atoms. In the case of a rigid model of the zeolite framework, this does not result in any deformation of the overall structure. The putative zeolite is studied with the objectives of (i) investigating a phenomenon and a tuning strategy that can be applied to materials allowing more flexible design approaches (e.g., MOFs and COFs, as mentioned in the introduction), where lateral pores can be tuned without any violation of chemical rules, and (ii) singling out an effect, the topology of the inner walls of microporous materials and degree of connectivity among cavities, that is usually overlooked in interpreting their intrusion/extrusion characteristics.

The key features of our computational setup are the following. ITT/mITT– and MFI/mMFI −water systems were simulated with the Bushuev-Sastre force field,^18,19^ which coincide with ClayFF^20^ in the case of rigid frameworks, and the SPC water model.^21,22,23,200^ Models with rigid zeolite frameworks correspond to the Kiselev model,^24^ which has been videly used for calculations of adsorption by zeolites. For flexible/charged models, subnanometer topological tuning has been achieved by inserting Lennard-Jones particles in lateral channels to prevent water penetration in these apertures. Additional calculations were performed for ITT-, MFI-, and TON-type pure silica zeolites immersed in water using the force field of Emami et al.^22^ for zeolites and the mW model for water^23^ to confirm the generality of our results, to confirm that our conclusions do not depend on the computational setup (see SI - TON is the shorthand for Theta-ONe zeolite, first synthesized by Barri et al.) We have run several constant pressure and temperature (300 K) molecular dynamics simulations of the duration of up to 15 ns per system using the DL_POLY^25^ and LAMMPS^26^ codes.

Previous analysis on the sensitivity of intrusion/extrusion characteristics to the parameters of the force field^27^ brought us to conclude that variation of point charges on Si and O atoms affect the hydrophobicity of (computational) zeolites. We set all partial charges on Si and O atoms to zero to make the putative pure silica ITT zeolite extremely hydrophobic. In the SI, we give extensive evidence that the conclusions of this work neither depend on the flexibility of the frameworks used to model the porous media, nor are affected by the absence of charges on the atoms. In the SI we provide the results of a detailed analysis of the implications of subnanometric topological tuning on energy applications of porous media.

The ITT/mITT computational sample consisted of a nanocrystallite containing a 3 × 3 grid of 18MR channels (Figure 1a) that, together with 50,000 water molecules, was placed in a cubic triperiodic box with an ∼12 nm edge (Figure S1). Though much smaller than particles used in experiments, such a nanocrystal contains the key elements of real crystallites: bulk (gray) and surface (orange) channels, the ones directly in contact with bulk water through 10MR windows. In the mITT case, due to the absence of lateral windows, all 18MR channels are the same; there is no difference between surface and bulk.

In Figure 2a, we report isotherms of ITT/mITT. The corresponding isotherms showing fractional loading are presented in Figure S5. Intrusion/extrusion pressure corresponds to a half loading. For ITT models, one notices that intrusion/extrusion follows a typical hysteretic trend, where the intrusion pressure, *P*_*int*_, largely exceeds the extrusion value,*P*_*ext*_, which makes it suitable for energy damping. In our simulations, ITT shows a sizable hysteresis, with *P*_*int*_ = 55 MPa and *P*_*ext*_ = 7 MPa, corresponding to a shock-absorber-like behavior. As extensively discussed in the literature, in both experiments and simulations, hysteresis is due to the presence of intrusion- and extrusion-free energy barriers, which keep the system kinetically trapped in a metastable state;^28,29^ that is, the system can escape from the metastable state on a time scale longer than the duration of the simulation or experiment. Since these barriers drop as pressure increases or decreases, depending on the process considered,^29−31^ when the pressure reaches a threshold that the barrier is comparable with the thermal energy, *k*_B_*T*, the system transits from the metastable to the stable state. Kinetic trapping is more severe for simulations than experiments given the shorter duration of the first (nanosecond) with respect to the second (seconds to minutes); hence, hysteresis in simulations is typically more pronounced than in experiments.

Within the accuracy of the simulation protocol characterized by 5–10 MPa pressure increments/decrements, upon modification from ITT to mITT, *P*_*int*_ and *P*_*ext*_ consistently increase by 20 MPa, with a minimal effect on the shape and the area of the hysteresis loop. Thus, the results presented in Figure 2a show that small lateral subnanometer pores can be used to control and tune intrusion/extrusion characteristics, increasing or decreasing *P*_*int*_ and *P*_*ext*_ depending on whether one opens or closes lateral apertures. It is remarkable that this can be achieved without altering other properties, such as the percent of hysteresis %_Δ*P*_ = (*P*_*int*_ – *P*_*ext*_)/*P*_*int*_ × 100, the chemistry of the material, and the geometry of the main pores.

To illustrate the broad relevance of our findings, we also considered the case of silicalite-1 (Figures 2b, S5b, and S6), a pure silica zeolite of MFI type. Crystallites of 2 × 2 × 3 unit cells were immersed in water (Figures S2 and S7), and the same simulation method, as in the case of ITT, was applied. Silicalite-1 presents molecular spring characteristics (small intrusion/extrusion hysteresis),^16,17,26,27^ which, together with its high ∼10 J/g energy density, make it suitable for mechanical energy storage. The modified MFI zeolite, mMFI, was obtained by closing all lateral windows of the [010] main channel by -Si–O–Si-chains (rigid/uncharged model) or by spherical particles (Figure S2b). Upon closure of lateral windows, silicalite-1 shows a ∼75 MPa increase of the intrusion (and extrusion) pressure (Figure 2b and Figure S6), regardless of the force field atomistic model (rigid/uncharged vs flexible/charged) used to represent zeolite/water interactions. This shift represents an ∼80% increase of the intrusion (extrusion) pressure with respect to the 96 MPa experimental value of the original structure. In the case of MFI/mMFI, the pressure shift is accompanied by an increase of hysteresis (Figure S10); however, this increase is moderate, and the system remains a molecular spring. The potential technological consequences of this intrusion pressure shift are discussed in detail in the SI.

We now focus on the microscopic mechanism responsible for the observed effect of lateral windows on intrusion/extrusion pressures. For ITT, the first surprising observation is that adjoined lateral windows get wet during the intrusion, while macroscopic theories^32,33^ suggest that smaller hydrophobic cavities (10MR) should get intruded at higher pressures than larger pores (18MR). We speculate that this unusual behavior is due to the reduced depth of 10MR windows: H_2_O molecules in 18MR channels can protrude through the thin lateral windows to form hydrogen bonds with water on the other side, water in other channels, or surrounding bulk water (see below), similar to the sagging suggested by Patankar for continuum liquids wetting shallow cavities.^34^ A second important observation is that intrusion of water in 18MR channels is correlated with the wetting of adjoined 10MR windows, as illustrated by the correlation between the number of water molecules in the bulk 18MR channel and those inside adjoined 10MR channels (Figure 3a—see also Movies S1 and S2). We remark that intrusion of 18MR channels does not occur by means of infiltration of water through lateral pores; rather, water in lateral pores facilitates the intrusion through the main 18MR channels. Though MFI presents a different structure than ITT, also in this case lateral apertures contain water molcule during intrusion, 0-2 in the case of ITT (Figure 3d) vs 0-3 in the case of MFI (Figure S7), confirming that the same mechanism is at the basis of the subnanometric topological tuning of both systems.

**Figure 3:**
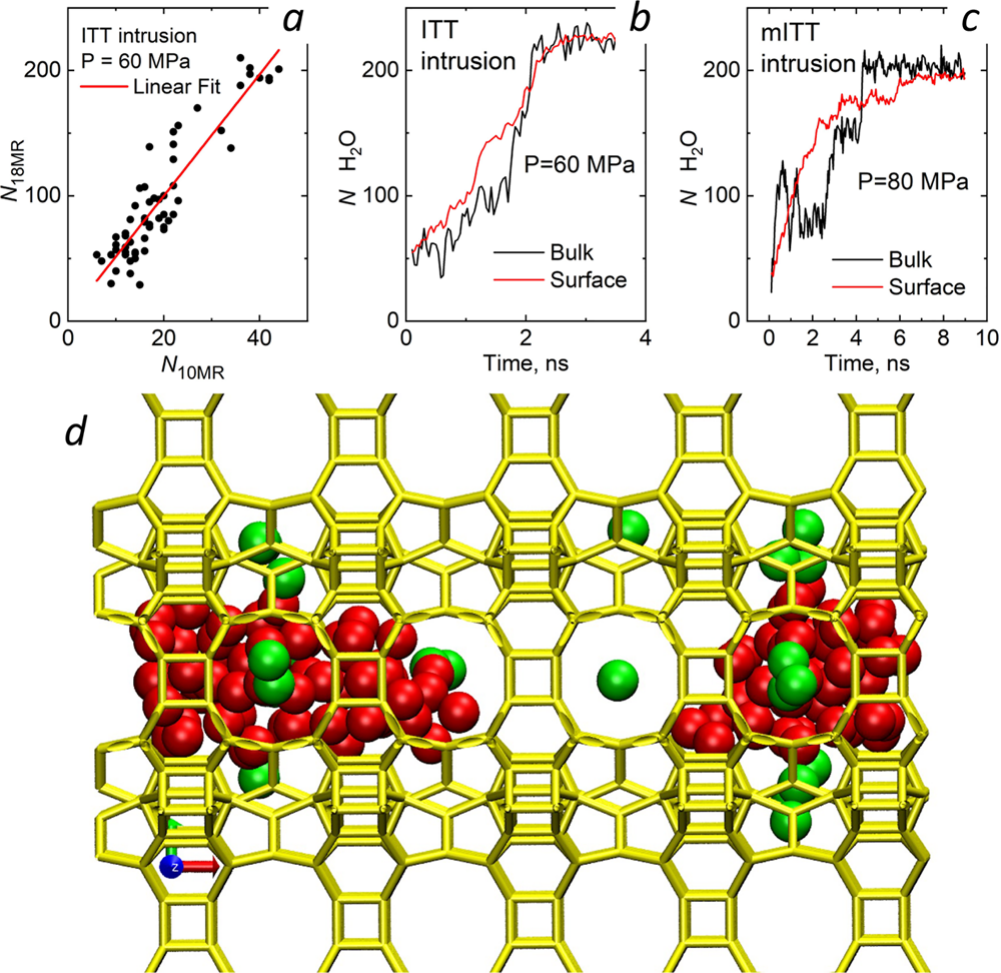
(*a*) Correlation between the number of water molecules inside 18MR and 10MR pores. Number of water molecules in the bulk and surface 18MR channels vs time for ITT (*b*) and mITT (*c*) at pressures near the corresponding *P*_*int*_. Values for surface channels have been obtained, averaging over the eight independent channels. (*d*) 18MR channel of ITT at intermediate intrusion. Color coding is the same as in Figure 1b.

An intuitive argument to explain why the wetting of lateral apertures eases intrusion has been alluded to above: the energetic cost of intruding hydrophobic channels is lowered by water forming hydrogen bonds across 10MR apertures with the liquid in neighboring 18MR (ITT) or straight 10MR (MFI) channels, if present, or the bulk liquid, for surface pores. This argument can be reformulated within the capillary theory of intrusion.^35^ Let us model 18MR channels by (solid) cylinders presenting lateral apertures. If 10MR windows remain empty during the intrusion, water entering in a channel has to pay an energy penalty corresponding to (i) the interface energy between the liquid and the solid part of the surface of the cylinder, *A*_*s*_*σ*_*sl*_, plus (ii) the surface energy of the intruding liquid in contact with empty 10MR adjoint apertures, *A*_*adj*_*σ*. Here, *σ*_*sl*_ is the solid–liquid interface energy, σ is the surface tension of water, and *A*_*s*_ and *A*_*adj*_ are the areas of the solid surface of the channel and lateral apertures. If water bridges with the liquid on the other side of 10MR windows, there is no interface energy penalty to be paid relative to the surface tension of water as there is no interface between water and air. In practice, bridging between water across 10MR windows reduces the hydrophobicity of the main channels, resulting in a lower intrusion-free energy barrier, making it easier for water to intrude the main channel. Following this macroscopic analysis (see details in SI), for ITT we determined an (overall) effective solid–liquid interface energy σ_*eff*_^*ITT*^ of ∼20 mJ/m^2^, corresponding to an effective contact angle θ_*eff*_^*ITT*^ = 108°.

The arguments discussed above allow rationalization of the higher intrusion/extrusion pressures of mITT over ITT. In mITT, where water bridging cannot occur, one expects the intrusion barrier to be higher. Consequently, in mITT, one needs to apply a higher pressure to force the liquid beyond this barrier to intrude into 18MR channels. During extrusion, the stabilization effect of water bridging results in a higher extrusion barrier in ITT than in mITT. Hence, in ITT one must force extrusion by reaching lower pressures. This is consistent with the capillary theory of intrusion/extrusion,^35^ which predicts higher intrusion/extrusion pressures for materials with higher (effective) surface tension, σ_*eff*_^*mITT*^ ∼ 26 mJ/m^2^ vs σ_*eff*_^*ITT*^ ∼ 20 mJ/m^2^, ∼25% higher in mITT because it lacks lateral apertures. This brings an increase of the contact angle of the 18MR cavity walls to θ_*eff*_^*mITT*^ = 114° (see the SI).

Summarizing, we found that by suitably designing features decorating the lateral walls of the main cavities of porous materials, in terms of both their width and depth, one can control the intrusion/extrusion pressure of lyophobic microporous materials without significantly altering the other properties of the system. For example, in ITT/mITT the hysteresis does not change while, though it changes for MFI/mMFI, the system remains a molecular spring. In particular, we observed a remarkable *P*_*int*_/*P*_*ext*_ shift of 20 and 75 MPa toward higher values upon closing the lateral windows of ITT and MFI frameworks, respectively. Subnanometric topological tuning is, hence, alternative or cooperative with other tuning strategies, such as modifications of the overall size of the cavities. Indeed, the effect of subnanometric topological tuning is surprising and conflicts with macroscopic criteria that, so far, resulted in being valid also at the nanoscale: corrugations and cavities decorating surfaces enhance the intrinsic hydrophobicity of materials.^28,36−38^ Our results show that when these cavities are particularly shallow, as in the lateral windows of ITT and MFI, the effect is inverted: cavities act, in a loose sense, as *“hydrophilic”* patches, reducing the hydrophobicity of material through hydrogen bond bridging when there is water on the other side of the apertures.

The presence of lateral apertures is also responsible for the peculiar intrusion mechanism of ITT. This is illustrated by comparing the time evolution of the number of molecules (*N*) in the surface and bulk 18MR channels of ITT/mITT and MFI/mMFI (Figure 3b and c; see also Figures S8, S9, S11–S13, and S17–S20). For surface channels, *N* is averaged over all the pores present in the crystallite. One notices that intrusion in ITT and MFI occurs first on the surface and then in the bulk channels. For mITT and mMFI, there is no preferred order for filling surface and bulk channels. The anticipated intrusion in the surface cavities of ITT/MFI is due to the water bridging across lateral apertures: the liquid entering in surface channels can always form water bridges with (and get stabilized by) the liquid surrounding the crystallite.

On the contrary, liquid intruding bulk channels can bridge across 10MR apertures only if the surrounding channels are already intruded (see Movies S1 and S2). In crystallites of experimental size, we expect that the enhanced intrusion associated with 10MR windows gives rise to an *avalanche mechanism*: water intrusion starts at the surface of ITT crystallites and advances toward their center. A more systematic study is in progress about the intrusion mechanism using free energy techniques.^39^ The relevance of this mechanism in actual porous materials with lateral apertures decorating the main cavities, for example, Cu_2_(tebpz) MOF, ZSM-57, DAF-2, DAF-1, ITQ-50, ZK-5 zeolites, and the ZIF-8 MOF and its derivatives, which have been investigated by Mortada et al.,^15^ will be investigated in future theoretical and experimental works.

MFI- and TON-type zeolites have 3D and 1D systems of 10MR channels, respectively. Thus, the topologies of mMFI and TON porous systems are similar, but the geometrical characteristics are different. We have performed simulations for both zeolites using a coarse-grained water model (mW).^23^ Experimentally, the difference between the intrusion pressures of TON and MFI is 94 MPa. Our simulations show a difference of 43 MPa (Figure S15). Considering silanol defects in silicalite-1 crystals, we conclude that the models give a reasonable result. Simulations of ITT with this force field show that water intrusion starts from surface channels (Figure S11). Thus, the effect observed for ITT and MFI systems does not depend on a force field or a simulation method. The shift of intrusion pressure of 75 MPa obtained for MFI/mMFI systems has experimental support.

In conclusion, in this work, we introduced a novel design strategy for tuning the intrusion (wetting)/extrusion (drying) characteristics of microporous materials by nonwetting liquids. We exploited the flexibility of atomistic simulations to disentangle the effects of the chemistry of materials and the geometry of their main channels from the characteristics of secondary subnanometric pores decorating the surface of the principal cavities. We have shown that lateral windows, typically neglected in the design and analysis of porous media characteristics, play a key role. Our findings have potentially a broad scope to interpret experiments and design porous material for energy applications, catalysis, liquid/liquid and liquid/gas separation, chromatography, nanofluidics, biology, and many more: all those research fields and technological applications where intrusion/extrusion and flowing of a liquid within a nanoconfining environment is a key process.^1−4,9−11^ The transformation of the general principles enunciated in this work into practical guidelines requires further understanding of the relation between the size and shape of lateral pores, their thickness (that here is limited to 1–3 atomic layers), and intrusion/extrusion pressure. Experimental studies are also needed to validate the theoretical principle discussed here and to develop methods to synthesize porous media of suitable chemistry and morphology. Work is in progress in these directions.

## Supporting Information Available

Simulation details, detailed analysis of the intrusion/extrusion characteristics of silicalite-1, additional figures, and capillary theory of intrusion (PDF)
nl1c02140_si_002: DL_POLY input files for ITT (ZIP)
nl1c02140_si_003: DL_POLY input files for mITT (ZIP)
nl1c02140_si_005: DL_POLY input files for MFI (ZIP)
nl1c02140_si_004: DL_POLY input files for mMFI (ZIP)
nl1c02140_si_006: DL_POLY input files for MFI with flexible force field (ZIP)
nl1c02140_si_008: DL_POLY input files for mMFI with flexible force field (ZIP)
Movie S1: Lateral view during water intrusion in three neighboring channels (MP4)
Movie S2: Top view during water intrusion in three neighboring channels (MP4)
