# Supplementary material for: Subnanometer Topological Tuning of the Liquid Intrusion/Extrusion Characteristics of Hydrophobic Micropores
Source: Nano Lett. 2022 Mar 8;22(6):2164–9. doi: 10.1021/acs.nanolett.1c02140 (PMC8949755; doi:10.1021/acs.nanolett.1c02140)
Supplement: Supplementary file 1 — nl1c02140_si_001.pdf [file nl1c02140_si_001.pdf]

Supporting Information:

Subnanometer Topological Tuning of the Liquid Intrusion–Extrusion characteristics of Hydrophobic Micropores

Yuriy G. Bushuev,*[a] Yaroslav Grosu,[b] Mirosław A. Chorążewski,[a] Simone Meloni*[c]

[a] Prof. Y. G. Bushuev,*, Prof. M. A. Chorążewski
Institute of Chemistry
University of Silesia in Katowice
Szkolna 9 street, 40-006 Katowice, Poland
E-mail: yuriy.bushuev@us.edu.pl, miroslaw.chorazewski@us.edu.pl.

[b] Prof. Y. Grosu,
Centre for Cooperative Research on Alternative Energies (CIC energiGUNE),
Basque Research and Technology Alliance (BRTA),
Alava Technology Park, Albert Einstein 48, 01510 Vitoria-Gasteiz, Spain
E-mail: ygrosu@cicenergigune.com.

[c] Prof. S. Meloni*
Dipartimento di Scienze Chimiche, Farmaceutiche ed Agrarie (DOCPAS),
Università degli Studi di Ferrara (Unife),
Via Luigi Borsari 46, I-44121, Ferrara, Italy; simone.meloni@unife.it

Simulation details.

The majority of computer simulation studies for adsorption in zeolites are based on the Kiselev approach,[1] which have shown a realistic approximation of experimental data for many systems.[2,3,4,5] According to the approach, zeolite atoms are fixed in their crystallographic positions. For decreasing a system's complexity, it was proposed to neglect van der Waals interactions with silicon atoms, which are shielded from adsorbate molecules by surrounding oxygen atoms.

The simplified guest-host potential function (Kiselev force field) contains a Lennard-Jones (**LJ**) term that acts between the oxygen atoms (O) of the framework host (H) and the atoms of the guest adsorbate (G) and an electrostatic term that acts between all charged atoms.[2]

$$U_{\mathrm{GH}}^{\mathrm{Kiselev}} = \sum_{\mathrm{G,H=O}} 4\epsilon^{\mathrm{GO}}\left\{\left(\frac{\sigma^{\mathrm{GH}}}{r_{\mathrm{GH}}}\right)^{12} - \left(\frac{\sigma^{\mathrm{GH}}}{r_{\mathrm{GH}}}\right)^{6}\right\} + \sum_{\mathrm{G,H\in T,O,M^+}} \frac{q^{\mathrm{G}}q^{\mathrm{H}}}{r_{\mathrm{GH}}} \quad , \text{(S1)}$$

where q is the charges of atoms.

Neglecting part of interactions for rigid zeolites reduces the computational cost significantly, allowing the simulation of large systems with the same computational resources.

We immersed tiny zeolite crystals in water (**Figure S1**). Previous investigations of **pure silica zeolites (PSZ)** – water systems demonstrated[6] a minor role of framework flexibility on intrusion/extrusion pressure which supports the Kiselev model. The present paper obtained *the main results* for rigid zeolite frameworks and specially tuned water - zeolite interactions. We counted Si-Ow interactions but neglected the electrostatic term (Eq. S1).

We have performed additional calculations for some systems with flexible frameworks and electrostatic interactions to validate our conclusions. The third type of our simulations is based on a coarse-grained water model and flexible zeolite frameworks. It critically reduces computer resource consumption for large simulated systems, including several hundred thousand atoms.

Force Fields: Many FFs were proposed for a compute simulation of zeolites.[7,8] The variety of FFs rather indicates their inferiority: no one FF reproduces all zeolites' properties. Another significant problem is the transferability of FF among existing zeolite topologies.[5,9]

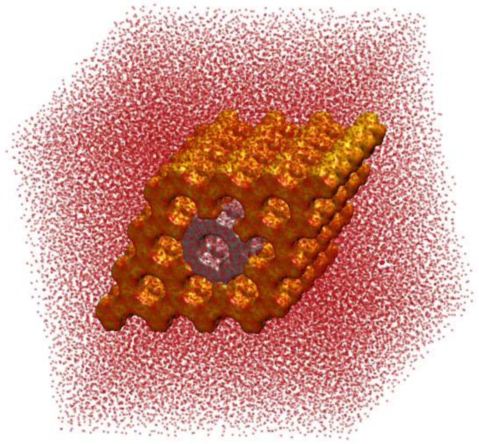

Figure S1. The simulation tri-periodic box containing the ITT crystal immersed in water.

We are based on the Bushuev – Sastre (BS) FF,[11] which have shown promising results in simulations of PSZs[12] and zeolite - SDA - water systems.[6,13] The BS FF is a modified ClayFF.[14] Partial atomic charges, LJ parameters in both FFs are the same. According to ClayFF, the $\varepsilon_{\mathrm{SiSi}}$ parameter of the LJ potential is several orders of magnitude smaller $\varepsilon_{\mathrm{OzOz}}$. In general, it corresponds to the Kiselev approach.

The BS and ClayFF coincide if frameworks are rigid. Additional terms are added to ClayFF for better corresponding to experimental structural and thermodynamic properties of actual zeolites when simulations are performed with flexible frameworks.

For comparison, some simulations we performed with other FF taken from the literature.[15] The parameters of the FFs are presented in Table S1. In both FFs, electrostatic interactions are always balanced by two- and three-body interactions to correspond to the experimental structure of zeolites.

Intercomponent (water - zeolite) electrostatic and van der Waals interactions are the main parameters defining the hydrophobicity of zeolites and, as a result, intrusion pressure. The electrostatic interactions in the system cannot be tuned largely due to their strong influence on zeolite and water structures. If a framework is flexible, variation of partial atomic charges influences the crystallinity of zeolite. Meanwhile, we can vary the charges if atoms are fixed in their crystallographic positions.

Table S1. Parameters of interparticle interactions

BS (modified ClayFF) + SPC			
	ε, J/mol	σ, Å	q, e
Si	0.077	3.30	2.1
Oz	650.2	3.166	-1.05
Ow	650.2	3.166	0.82
Hw	—	—	-0.41
Three body	k, kJ/(mol rad^2)	Θ_0, deg	
Oz-Si-Oz	121.71	109.47	
Si-Oz-Si	135.1	142.0	
Surface Model[15] + mW			
	ε, J/mol	σ, Å	q, e
Si	389.1	3.742	1.1
Oz	225.9	3.207	-0.55
Oz-Ow	1040	3.30	
Si-Ow	0.91	3.234	
Two body	k, kJ/(mol A^2)	r_0, A	
Si-Ow	2385	1.5	
Three-body	k, kJ/(mol rad^2)	Θ_0, deg	
Oz-Si-Oz	836.8	109.5	
Si-Oz-Si	836.8	149.0	

The second choice that drastically influences the results of simulations is the water model. The structure of a molecule, partial charges, van der Waals interactions are varied to a large extent but balanced again. The main results of our work were obtained with the SPC water model.[16] Some supporting simulations were performed with mW water model.[17] The mW mimics the hydrogen-bonded structure of water by introducing a non-bond angular dependent term that encourages tetrahedral configurations. A water molecule has only one uncharged site for intermolecular interactions.

The strategy of results validation. To validate our results, we have performed a set of simulations

1. Rigid ITT-, MFI-type and modified (mITT, mMFI) frameworks with zero partial charges on zeolite atoms (the Kiselev-type FF with tuned host-guest interactions). These are **rigid/uncharged** models;
2. The flexible MFI/mMFI frameworks with charged zeolite atoms and a limited set of simulations for ITT/mITT. These are **flexible/charged** models;
3. The flexible MFI, ITT, and TON frameworks with charged atoms and the mW water model (Table S1). These are **coarse-grained** models.

We compared calculated intrusion isotherms for MFI and TON zeolites with available experimental data. The TON-type zeolite has 1D straight 10MR channels. There is a similarity between mMFI and TON frameworks. Comparison of isotherms demonstrates additional support to our conclusions.

The advantage of rigid uncharged structures is the opportunity to close some pores by -Si-O-Si- chains, the same atoms that form channel walls. The valence state of atoms and the direction of bonds are not valuable because all atoms are in fixed positions. These models highlight the effect of changing topology on intrusion/extrusion pressure.

For blockage of lateral pores in the flexible model, we used additional LJ atoms in fixed positions that interact only with water molecules. We mechanically prevented water penetration in some pores and meanwhile did not change vibrations of zeolite atoms. However, their interactions with water, to some extent, influence results. We have additional degrees of freedom in the particle - water parameters of LJ interactions.

Preliminary ITT – water simulations using the flexible/charged model have shown that zeolite adsorbed water at small pressures, and extrusion occurs only at negative pressures. The ITT framework contains extra-large (among zeolites) 18MR channels, and according to the Laplace law, low intrusion pressure is expected.

Our goal is to investigate the effect of topological tuning of porous materials. ITT-type zeolite has the largest straight main channels, which smaller ones interconnect. This microporous material with an aperture of 1.53 nm is close to a border (2 nm).with mesoporous ones. For example, mesoporous amorphous silica materials grafted by chlorodimethyloctylsilane have diameters of pores: 2.68 (MCM-41), 3.08 (HMS), and 4.3 (SBA-15) nm.[18]

We believe that the conclusions of this work hold for other classes of porous materials like grafted mesoporous silica, MOFs, COFs. We investigated artificial model systems. To shift the intrusion-extrusion isotherms, we have tuned water - zeolite interactions making the zeolite more hydrophobic. It was reached by putting all silica charges to zero in the models with rigid frameworks. It was reported[18] that cylindrical pores of SBA-15, depending on synthesis temperature, can be connected by a secondary network of smaller micropores. The interaction of water with highly hydrophobic organic molecules covered silica walls are smaller than with silica. Thus, our simulations with uncharged silica atoms mimic actual systems.

We have performed calculations for MFI zeolite, which has only 10MR channels, to demonstrate the topological tuning effect for the typical microporous materials. Using the smaller systems than in the ITT case, we calculated intrusion/extrusion isotherms for rigid/uncharged and flexible/charged models. Verification of our approach is presented below.

Methods of simulations.

We have performed molecular dynamics simulations of zeolites immersed in water using a constant number of particles, pressure, and temperature (NPT-ensemble). Nosé – Hoover thermostat and barostat with Melchionna[19] modification of the Nosé – Hoover algorithm with a relaxation time of 1 and 5 ps, respectively, was applied. Simulations were performed at T=300 K and a wide range of pressures, using the DL_POLY version 4.09 and 4.10 molecular dynamics code. Equations of motion were solved numerically by the velocity Verlet algorithm with a time step of 1 fs for flexible frameworks or 2 fs for rigid frameworks.

Simulations were performed with the BS+SPC FFs presented in Table S1. Lorentz-Berthelot combination rules were used to calculate LJ parameters of water – zeolite interactions. The interaction models and computational approach have been successfully used in previous water intrusion/extrusion in/from zeolites studies.[6] For the systems with rigid frameworks, partial charges on Si and O atoms were set to 0.0 *e* (rigid/uncharged models).

Our computational sample is a cubic periodic box containing a 4×4×5-unit cells crystallite of ITT-type zeolite and 50,000 water molecules, as presented in **Figure S1**. When the adopted model had a rigid framework, we used –Si-O-Si- chains placed in 10MR windows to produce modified ITT (mITT). In the case of flexible/charged frameworks, lateral 10MR channels were closed by LJ particles (**Figure S2**), placed in the centres of 10MR channels, interacting only with water molecules with ε = 650.2 J/mol and σ = 5.5232 Å.

For silicalite-1 and its modified counterpart, the periodic box contained a 2×2×3-unit cells (1152 SiO_2) crystallite of MFI-type zeolite and 10,000 water molecules. We used different methods of pore closing for the rigid/uncharged and flexible/charged MFI models. We added –Si-O-Si- chains bridging atoms on the opposite sides of lateral windows in the case of the rigid framework. This strategy cannot be applied to the flexible/charged case, where we must preserve the flexibility and charge neutrality of the zeolite framework. The former requires that the coordination number of Si and O atoms be preserved. For the flexible/charged case, lateral 10MR sinusoidal windows decorating the *walls* of [010] channels have been closed by inserting LJ particles at fixed positions (**Figure S2**). These additional particles interact only with water molecules: ε = 650.2 J/mol and σ = 3.166 Å.

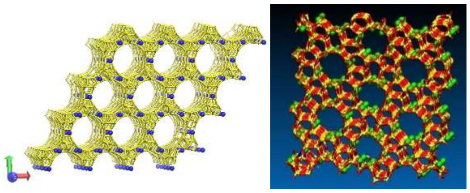

Figure S2. Modified ITT (left) and MFI-type (right) zeolites. Balls represent LJ particles closing the 10MR channels of flexible/charged models of zeolites.

Compressed directories containing CONFIG, FIELD, CONTROL files for the DL_POLY program are provided in the Supporting Information.

Electrostatics is computed resorting to the well-established Smooth Particle Mesh Ewald (SPME) method, computing the short-range part of the interaction in the real space, till a 9 Å cut-off, and the long-range part in the reciprocal one. This setup has been already validated in the literature.[20] All van der Waals interactions are computed in the real space with a 9 Å cut-off, corresponding to the typical ~2.5 σ cut-off value. So-called long-range corrections, based on the pair correlation function, were applied to the potential energy and the pressure of the system.

PSZ - water systems (MFI, TON, ITT) with the coarse-grained mW water model were simulated in NPT-ensemble, using the LAMMPS code.[21,22] Temperature and pressure were controlled using a Nosé – Hoover thermostat, with a 0.25 ps damping constant, and a barostat, with a damping parameter of 1 ps, at a temperature of 300 K. Surface Model FF (Table S1) was applied to calculate zeolite atoms interactions. The LJ parameters of Ow – O and Ow – Si interactions are: ε = 1040 J/mol, σ = 3.3 Å, and ε = 3.8 J/mol, σ = 3.234 Å, respectively. The time step equals 5 fs as recommended in the original papers.[17,23,24] The real space cut-off was 11 Å. A particle-particle particle-mesh (pppm/sg) solver was used to compute long-range interactions.

The simulation boxes contained 3×3×5-unit cells of MFI and 3×3×10-unit cells of TON PSZ, and 30000 water molecules (mW), 4×4×5-unit cells crystallite of ITT-type zeolite and 50,000 water molecules (mW).

Results.

Selection of force fields parameters.

The problem of zeolite – adsorbate parameters choice is discussed in the literature.[2,25] In the case of water, it was demonstrated (see SI[6]) that intrusion pressure depends to different extents on the flexibility of a framework, partial charges on zeolite atoms, and parameters of LJ potentials.

The main object of our investigation is the zeolite–water system with extra-large 18MR straight channels (ITT) interconnected by smaller 10MR channels. There is no experimental data about intrusion/extrusion isotherms for the system. Computations with the original BS+SPC and coarse-grained FFs were shown that ITT zeolite adsorbs water at zero pressure and extrudes it only at negative pressures.

Simulations with the mW model using the LAMMPS code demonstrate ca. 100-time faster productive runs than simulations with the BS+SPC FF (flexible/charged). However, the code cannot directly work with rigid frameworks or frozen atoms in NPT-ensemble. Thus, simulations of mITT or mMFI with LAMMPS code can be performed only using other methodology when a moving piston creates pressure in the simulation box.

The productivity advantage allows tuning parameters of guest-host interactions using well-established intrusion isotherms for MFI and TON-type zeolites. Repeating the simulations with a set of parameters, we tried to get isotherms close to the experimental ones. Results are presented in **Figure S3**.

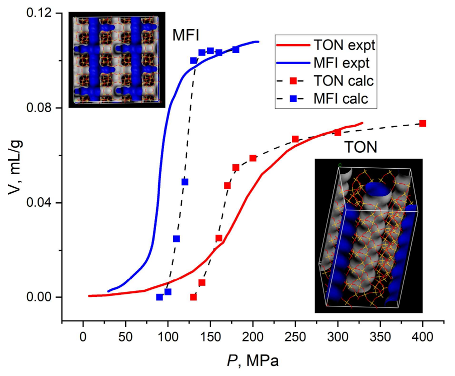

Figure S3. Experimental[26,27] and calculated intrusion isotherms for MFI and TON-type PSZ. *V* is the volume of intruded water (mL per 1 gram of zeolite), *P* is pressure. The coarse-grained model was used.

Taking the parameters of water - zeolite interactions, we have repeated calculations for ITT-type zeolite and found adsorption at P=0 MPa. Thus, two different calculations for flexible/charged and coarse-grained models demonstrate the small hydrophobicity of ITT. It is possible to investigate intrusion/extrusion at negative pressures. However, firstly it is far from the experimental conditions, and secondly, the properties of stretched water are different from compressed water: cavitation is expected.

We used another strategy of simulations. Electrostatic zeolite–water interactions can be tuned to make zeolite more hydrophobic. However, the range of tuning is limited. These interactions are balanced by two- and three-term interactions. There is no possibility to vary partial charges of Si and O atoms without changing the zeolite structure. Charges on atoms in both models used in our investigations are different. The easy way to make ITT more hydrophobic is to employ a rigid framework neglecting electrostatic interactions (rigid/uncharged models). In this case, computational costs are much lower, and large systems can be simulated using the same resources.

Silicalite-1 (MFI) was a testing system in many investigations. **Figure S4** demonstrates some isotherms obtained with different methods of calculations and force fields. Orange, olive and red curves show our results. The results obtained for the flexible/charged model (the orange curve) mean that the hydrophobicity of silicalite is underestimated. At the same time, two other isotherms (olive and red curves) are close to the experimental one. It was shown[28] that intrusion pressure in silicalite-1 is susceptible to the quality of the material. Silanol groups make it more hydrophilic. The ideal pure silica silicalite-1 without crystal defects used in the models must be more hydrophobic than the actual material.

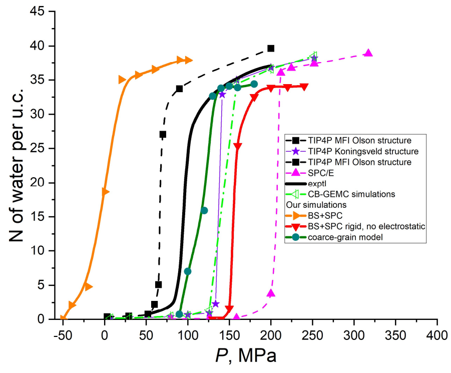

Figure S4. Experimental[28] and calculated[5,29] intrusion isotherms for MFI. Our calculations are: BS+SPC flexible with electrostatics (flexible/charged) shown by orange; rigid framework without electrostatic (rigid/uncharged) is designated by red colour; the olive curve represents the system with the coarse-grained mW model.

Thus, based on the previous considerations, we may expect slightly higher hydrophobicity for ITT-type zeolite than for the actual material if we select the rigid/uncharged model.

Remarks about data presentation

Results of computer experiments, the intrusion/extrusion isotherms can be presented differently. It may be the volume of the intruded liquid per gram of zeolite (**Figure S3**) or the number of molecules per unit cell vs pressure (**Figure S4**). To compare different systems and highlight only the process of intrusion/extrusion, we computed the fraction of water in the zeolite as a function of pressure. The fractional loading, $\chi(P)$, defined as the ratio between the average number of water molecules within the crystal at pressure P over the value at the maximum pressure - the saturation loading: $\chi(P) = N(P)/N(P_{max})$. Depending on our task, we present isotherms differently.

In addition to the Figures presented in the main text, **Figure S5** shows fractional loadings for both types of zeolites ITT and MFI for rigid/uncharged models. By definition, intrusion/extrusion pressure is measured at χ=0.5. In both cases, we see a shift of isotherms towards higher pressure when lateral pores are closed.

In the main text and the SI, we present kinetic curves showing variations of the number of water molecules in the zeolite channels during intrusion or extrusion. These values depend on the length of channels of the crystal grain used for simulations.

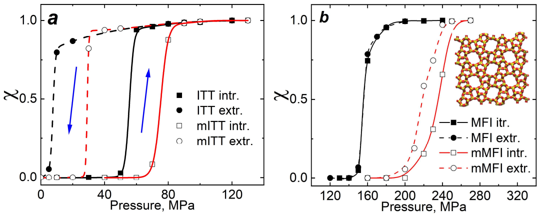

Figure S5. χ-P graph for the intrusion/extrusion cycle of a) ITT/mITT and b) MFI/mMFI zeolites.

Silicalite-1

ITT/mITT systems are discussed in the main text. We performed intrusion/extrusion *in silico* experiments with and without electrostatic interactions between the solid and the liquid with rigid and flexible zeolite. Here we highlight the results for MFI/mMFI rigid/uncharged and flexible/charged models. These simulations are performed for much smaller systems than ITT/mITT.

To illustrate the generality of the subnanometric topological tuning, we considered silicalite-1, a PSZ of MFI type (**Figure S2**). The silicalite-1+water system was shown experimentally to be a molecular spring,[28,30], i.e., it demonstrates a small hysteresis (5.2%), returning, upon extrusion, ~94% of the energy stored in the loading cycle.[31]

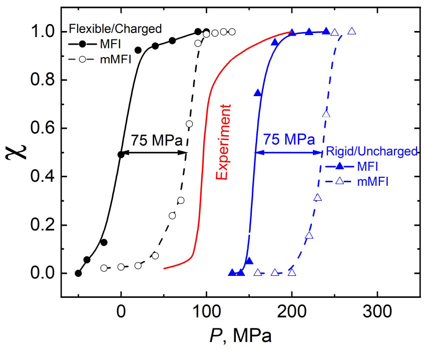

Figure S6. Intrusion isotherms for MFI- and mMFI-type zeolites. Simulations were performed for flexible and rigid frameworks with and without electric charges on Si and O atoms.

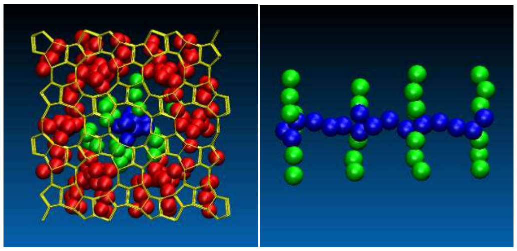

Figure S7. Water molecules in MFI-type zeolite: blue spheres are water in the bulk (central) channel; green spheres are in the adjoined sinusoidal channels; red spheres are in the surface channels.

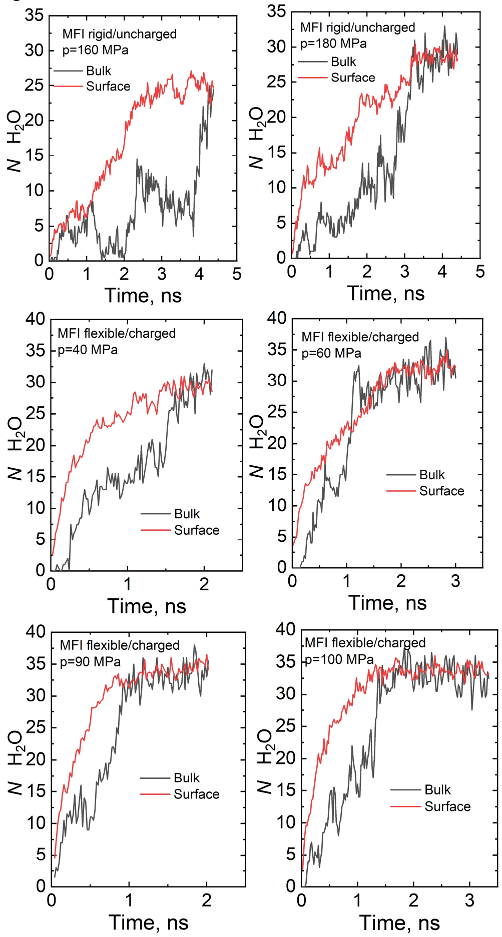

Figure S8. Kinetics of water intrusion in MFI-type zeolite.

In both cases, we found a small intrusion/extrusion hysteresis (**Figure S5b**), with charges and flexibility acting only on the absolute value of the intrusion/extrusion pressure, namely making silicalite-1 hydrophilic, with $P_{int} \sim P_{ext} \sim 0$ MPa as compared with $P_{int} \sim P_{ext} \sim 160$ MPa for the rigid/uncharged case. These values must be compared with $P_{int} = 96$ MPa and $P_{ext} \sim 91$ MPa experimental values (**Figure S6**).

We have investigated the wetting kinetics to highlight the mechanism of water penetration in MFI and mMFI. For this purpose, three types of water molecules were selected depending on their positions in the crystal, as presented in **Figure S7**. Regarding the number of molecules in sinusoidal channels, we can conclude that there is no significant difference with the case of ITT.

There are 0 - 3 water molecules in the channels connecting 10MR straight channels of MFI (**Figure S7**), whereas 0 - 2 molecules in 10MR channels of ITT.

When we are counting molecules, half of them in the adjoined sinusoidal channels (green) are considered to belong to the bulk channel (blue) and a half to surface channels (red). At each history file time step, a number of water molecules in the bulk channel and an average number of molecules per one surface channel were calculated. Kinetic curves for MFI presented in **Figure S8** demonstrate a process of channel wetting. In the case of an actual cristal, we may expect water penetration to start from the surface channels and propagate to the bulk. For mMFI (**Figure S9**), all channels are in the same condition, and penetration can start from any channel randomly.

In the rigid/uncharged and the flexible/charged cases, the subnanometric topological tuning results in shifting the intrusion/extrusion pressures by ~75MPa, ~80% above the experimental value of the original structure.

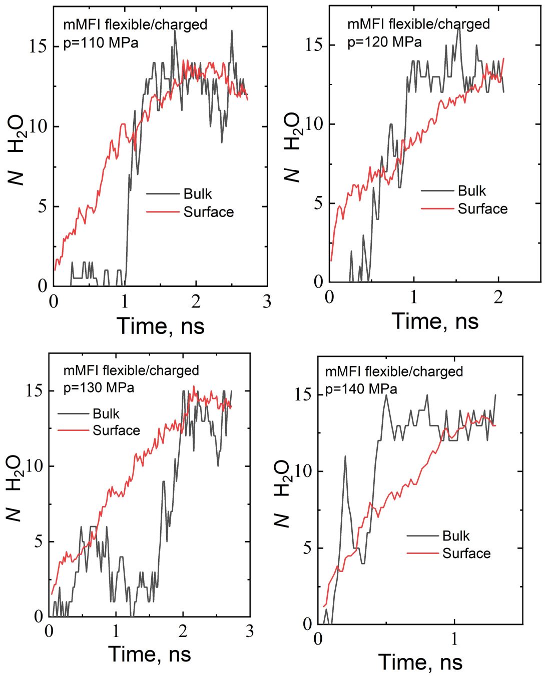

Figure S9. Kinetics of water intrusion in mMFI-type zeolite

It is interesting to compare the results obtained with the topological tuning against those that could be achieved with other well-established approaches, e.g., by changing the size of the main channels of porous media. Let us estimate how much the pore radius of a supposedly cylindrical channel-like structure must be changed to achieve a 75 MPa shift toward higher pressures. We base the following analysis on the Young–Laplace equation:

$$P_{int} = -2\gamma \cos\theta / r, \qquad \text{(S2)}$$

where $\gamma = 72.8$ mN/m is the water surface tension, θ is the Young contact angle of the material, and r is the radius of a cylindrical channel-like pore. In this semi-quantitative analysis, for silicalite-1, we used $r = 3.2$ Å.

Admittedly, the Young–Laplace equation and the cylindrical channel-like model of silicalite-1 are simplistic approximations for predicting/ characterizing the intrusion pressure of this complex porous material; However, here, the objective is only to obtain a rough estimate of the variation of the pore size to obtain a shift in the intrusion pressure comparable with the one obtained by the subnanometer topological tuning strategy, rather than rigorous quantitative analysis.

The effective Young's contact angle is estimated from Eq. S2, and the experimental value $P_{int} = 96$ MPa: $\theta = 102°$, consistent with the value of ITT discussed in the main text. Eq. S2 is also used to estimate the radius of porous systems with the same chemical characteristics, i.e., $\theta = 102°$, and a 75 MPa higher intrusion pressure, $P_{int} = 171$ MPa: $r = 1.8$ Å, i.e., to achieve the same shift in the intrusion pressure, the radius of the pore must be reduced by 1.4 Å.

This suggests that though in this work we are not proposing a specific synthetic strategy for closing lateral pores in zeolites, MOFs, COFs, or other porous systems, which is beyond the scope of the article and for which work is in progress, achieving the equivalent results with traditional strategies is very challenging. This makes it very worth considering the alternative tuning strategy suggested here and stresses the relevance of subnanometer topological features of a porous system in the analysis of their intrusion/extrusion characteristics.

Indeed, on top of the challenge of synthesizing a porous material of significantly different sizes, changing the radius of porous systems alters significantly other properties, e.g., hysteresis, as shown experimentally[32] and theoretically explained on the basis of the confined classical nucleation theory.[33] Our simulations show that subnanometer topological tuning can also affect hysteresis to some extent, but the changes are small/moderate, ~10 % with respect to the experimental intrusion pressure $\left(P_{int} - P_{ext}\right)/P_{int}^{exp} \times 100$), which still makes the modified silicalite-1 a molecular spring (putative) zeolite.

Concerning hysteresis, a final remark is in order. Both in experiments and simulations, hysteresis is a non-equilibrium effect due to the kinetic trapping of the system in a metastable state, e.g., the extruded state when the stable state is the wet pore and *vice versa*. In other words, at a given pressure between P_{int} and P_{ext} the system is either in the intruded or extruded states, depending on the initial condition.

The kinetic trapping is due to the presence of free energy intrusion and extrusion barriers requiring a characteristic time $\tau = \tau_0 \exp[\Delta G^\dagger / k_B T]$ to be overcome, where $\Delta G^\dagger$ is the free energy barrier, $k_B T$ is the thermal energy at the operative conditions and τ_0 is a pre-exponential factor setting the timescale of the process.[34,35,36] When simulations and/or experiments are shorter than τ, hysteresis manifests itself: the system remains in its original state through the (thermodynamically) stable one is the other. This condition ceases when the pressure reaches a threshold value where the barrier is small enough that τ is comparable/shorter than experimental or simulation time. Of course, since the timescale of simulations is many orders of magnitude shorter than the experimental/applicative value, nanoseconds *vs* seconds or minutes, the experimental hysteresis of a porous material subjected to the subnanometric topological tuning is smaller than the one predicted by simulations, e.g., the one shown in **Figure S10**. Thus, the theoretical hysteresis of **Figure S10** is an upper bound of the experimental hysteresis of the putative modified silicalite-1.

Though non-trivial, we attempt to analyze the energy density effects of subnanometer topological tuning in silicalite-1. As a first remark, we stress that for silicalite-1, we did not perform any fine subnanometer tuning. Rather, as for ITT, we closed all the lateral apertures of [010] channels, including the larger windows of a size comparable with the main cavities. These larger lateral windows allow access to sizable cavities; thus, at variance with ITT, for silicalite-1, this topological tuning is accompanied by a large ~45 % reduction of the pore volume available to the liquid. We remark once more that this large accessible pore volume is the result of drastic, sub-optimal tuning, adopted to allow a simpler comparison with the ITT/mITT, which has a different structure. Taking into account both the change of the intrusion pressure and accessible pore volume, the percentual variation of the energy density that can be mechanically stored in a porous material via liquid intrusion is given by

$$\%_{\Delta E} = \frac{(P_{int}^2 V^2 - P_{int}^1 V^1)}{P_{int}^1 V^1} \times 100 = (x_P x_V - 1) \times 100 \quad \text{(S3)}$$

where $x_P = P_{int}^2 / P_{int}^1$ and $x_V = V^2 / V^1$ and P^n and V^n are the intrusion pressure and the specific volume of the pores accessible to the liquid of system n.

For ITT/mITT, the increase in intrusion pressure $x_P = 1.4$ is accompanied by a reduction of the intruded volume $x_V = 0.875$, resulting in an $\%_{\Delta E} = 22.5\%$ increase of the stored energy. For MFI/mMFI we considered several scenarios. For example, MFI/mMFI behaves according to the predictions of the flexible/charged setup, with the intrusion pressure passing from ~0 to ~75 MPa, subnanometer topological tuning turns a material that cannot be used for energy storage into a molecular spring storing energy at moderate pressures, with a nominal infinite value of $\%_{\Delta E}$. If the same variation of the intrusion pressure and the specific volume of the pores accessible to the liquid is observed for a system with the experimental value of the reference intrusion pressure, 96 MPa, the shift of the operative pressure is accompanied by a 5% reduction of the energy density. In this case, establishing whether the topological tuning is convenient or not depends on the operative pressure of the mechanical energy source. Finally, if the intrusion pressure is the one determined for the rigid/uncharged silicalite-1 zeolite, the gain in the increase of the intrusion pressure is largely overwhelmed by the reduction of the specific volume of the pores accessible to the liquid, resulting in a severe reduction of the energy density storable in the porous system.

As we have remarked already several times, the very large reduction of the specific volume of the pores accessible to the liquid depends on our choice to close both large and small lateral apertures of MFI, to apply the same drastic tuning strategy of ITT, to transform a 3D into a 1D porous system. However, the optimal tuning strategy depends on the porous system at hand and how to implement it, whether lateral windows can be opened/closed, for example, by modifying linkers of MOFs and COFs (consider, e.g., the ZIF MOF family[37,38])

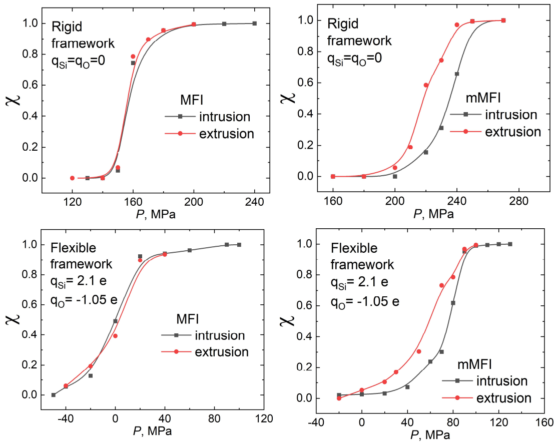

Figure S10. Intrusion-extrusion cycles for MFI- and mMFI-type zeolites. Simulations were performed for flexible and rigid frameworks with and without electric charges on Si and O atoms.

Supporting simulations of ITT

Coarse-grained model. Our simulations of the ITT-water system with the flexible framework and the coarse-grained water model (see Table S1) show the hydrophilicity of zeolite. It adsorbs water at P=0 MPa. We consider that a simulation of intrusion/extrusion processes at negative pressure has no physical sense. However, the calculated kinetic curves showing the loading of ITT channels with time give us information about the sequence of filling bulk and surface channels of zeolite. They are presented in **Figure S11.** At P=40 MPa, channels are filled by water fast.

Meanwhile, at P=0 MPa the system reaches saturation only after 10 ns. We see that surface channels are filled faster than bulk in both cases. The same behavior is observed for the system discussed in the main text (BS+SPC, rigid/uncharged). This

support our conclusion about the avalanche mechanism of ITT wetting at high pressure. Intrusion starts from surface channels and spreads through the crystal.

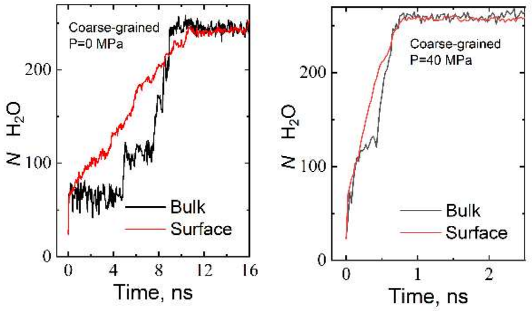

Figure S11. Kinetics of water intrusion in ITT-type zeolite. *N* is the number of water molecules in the bulk channel or the average number of molecules in the one surface channel.

BS+SPC, flexible/charged model. We have estimated the computational time needed for three simulation protocols. The rigid/uncharged model consumes ca. 10 times more CPU time than the coarse-grained model and ca. 10 times less than the flexible/charged one.

It is challenging to estimate intrusion pressure for reasonable computation time. We have tried to do it, but close to intrusion pressure, the production run exceeds 10 ns, as is demonstrated in **Figure S11**.

Kinetic curves for ITT and mITT are presented in **Figures S12 and S13.** Calculated isotherms presented as the fractional loading are shown in **Figure S14**. ITT at *P*=0MPa and mITT at *P*=20 MPa are not fully relaxed. We expect that both zeolites will be filled with water. As in the case of MFI, the inclusion of framework flexibility and electrostatic water interactions with zeolite only shifts intrusion pressure toward smaller values. However, the difference in pressure for ITT and mITT is 20 MPa in both cases.

Thus, we can conclude that the observed effect of topological tuning does not depend on variations of force field parameters or flexibility or rigidity of zeolite frameworks. Kinetic curves calculated for the model with coarse-grained water (mW) are similar to those obtained for more complex force fields. We believe that after additional tests, it opens a route to fast simulations of large systems with a reasonable consumption of computational resources.

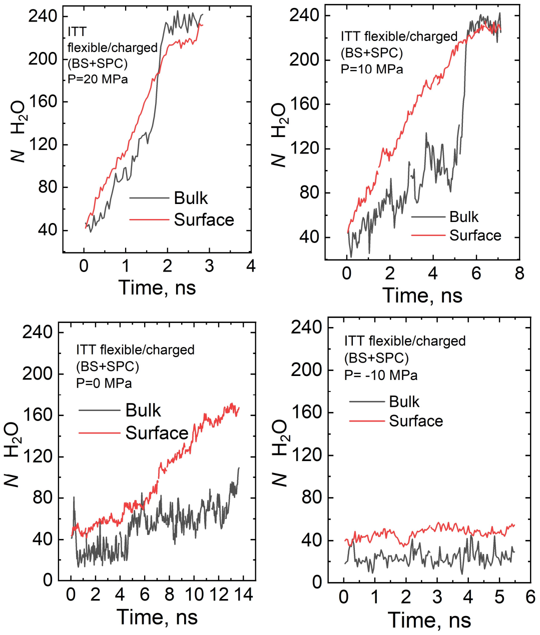

Figure S12. Kinetics of water intrusion in ITT-type zeolite simulated according to the flexible/charged model.

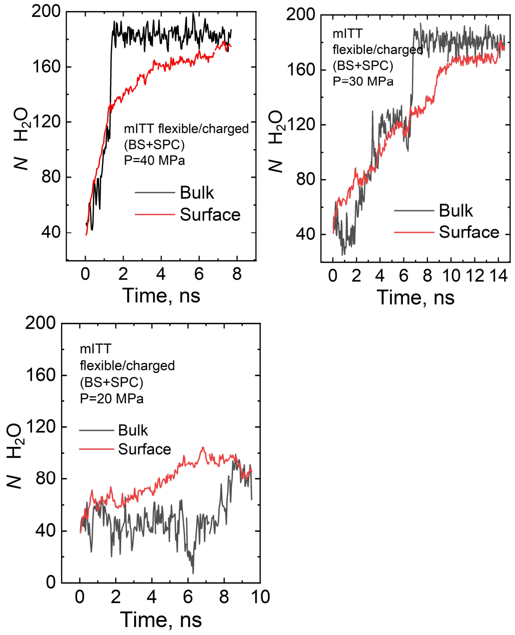

Figure S13. Kinetics of water intrusion in mITT-type zeolite simulated according to the flexible/charged model.

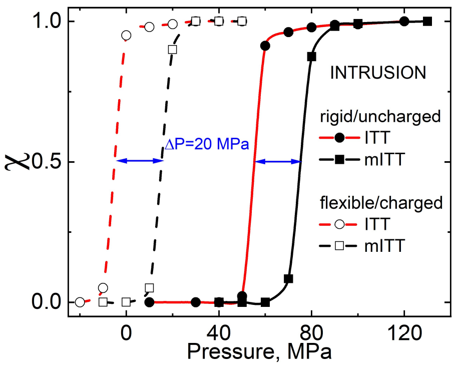

Figure S14. Intrusion isotherms for ITT- and mITT-type zeolites. Simulations were performed for flexible and rigid frameworks with and without electric charges on Si and O atoms.

Experimental support to our results

The main idea about the valuable role of lateral porosity on the energetic performance of lyophobic materials and non-wetting liquids has experimental support. Both MFI and TON topologies have only 10MR channels, but in TON, all channels run in one direction, and we have a 1D system of channels. Closing sinusoidal channels in MFI, we transform it to the 1D system. However, the shapes of the channels in mMFI and TON are different. The calculated and experimental results presented as loading fractions vs pressure for both PSZs are shown in **Figure S15.** Other presentations of data previously were shown in **Figure S3**. The difference of intrusion pressures measured experimentally is 94 MPa. Simulations with the mW model show the difference of 43 MPa. This value calculated for MFI and mMFI is about 75 MPa. The observation does not provide direct proof of our conclusions, but as a minimum, do not contradict the experiment.

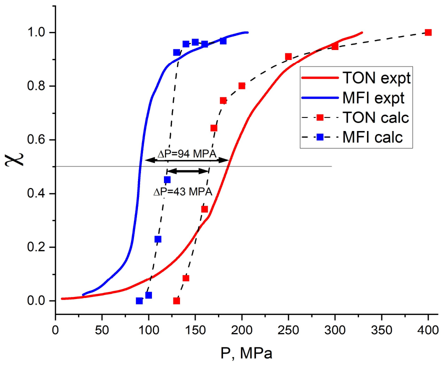

Figure S15. Experimental and calculated intrusion isotherms for MFI and TON.

Capillary Modelling model of water intrusion in zeolites

The hydrophobicity of material is conveniently described in terms of its surface tension with respect to a given liquid, σ_{sl}, which measures the energy penalty to put a solid surface of area 1 m^2 in contact with the given liquid. For a solid surface of area A the energy penalty of the interface, E_i, is

$E_i = A\sigma_{sl}$ (S4).

For complex materials, presenting surface corrugations or cavities in the crystal structure, one can introduce an effective surface tension, σ_{eff}, representing the energy penalty associated with wetting a surface of a nominal area A_{nom}:

$E_I = A_{nom}\sigma_{eff}$ (S5).

In other words, A_{nom} is the area corresponding to the footprint of the solid-liquid interface. The effective surface tension is an alternative way to formulate the well-known phenomena of enhanced hydrophobicity induced by wet (Wenzel[39]) and dry (Cassie-Baxter[40]) surface corrugations. From the effective surface tension, one can determine a corresponding effective contact angle $\cos\theta_{eff} = (\sigma_{sg} - \sigma_{eff})/\sigma_{lg}$, which, considering that for water it is usually $\sigma_{sg} \ll \sigma_{eff}$, can be replaced by the approximate formula

$\cos\theta_{eff} \sim -\sigma_{eff}/\sigma_{lg}$ (S6),

In the following, we will use $\sigma_{lg} = 65$ mJ/m^2, the surface tension of SPC water.[41]

Surface tension is a macroscopic concept stemming from the assumption that in a multiphase system, the difference between its energy, or the suitable thermodynamic potentials, and the bulk value of its components is proportional to the contact area between pairs of phases:

$E = \sum_{\alpha=1,N} E_B^\alpha + \sum_{\alpha>\beta} \sigma^{\alpha\beta} A^{\alpha\beta}$ (S7)

where E_B^α is the bulk energy (thermodynamic potential) of phase α, while $\sigma^{\alpha\beta}$ and $A^{\alpha\beta}$ are the surface tension and area, respectively, between phases α and β. Eq. S6 is usually denominated the sharp interface model of a multiphase system. Other terms, such as line tension τ, the energy cost to pay or reward to gain for the presence of a solid-liquid-gas triple line of length 1 m, might play an important role at the nanoscale.[42,18,43] Line tension is discarded in our analysis as the length of the triple line remains constant along with intrusion (see below). Other terms might also be important for determining an accurate value of the energy (thermodynamic potentials), such as the Tolman length[44,45], which determines the actual surface tension when a meniscus has a high curvature. However, we remark that this analysis is aimed at rationalizing microscopic results in terms of a language palatable to a broader audience, and these details can be discarded for the aim of the present short communication. It must also be remarked that this simple approach has already been tested for nanoscale porosities, giving surprisingly good results, semi-quantitative agreement with atomistic simulations.[35,36,46,47]

In the following, we will estimate σ_{eff} and θ_{eff} of ITT and mITT from A_{nom} of 18MR channels and the intrusion pressures measured in MD simulations. To this end, we model ITT and mITT 18MR channels as solid cylinders. The capillary theory of intrusion is conveniently written in terms of the grand potential, in which, considering that the solid cylinder does not change along with intrusion (rigid solid, as in the simulations), the bulk term of Eq. S6 can be written as $\sum_{\alpha=1,N} E_B^\alpha = -\Delta P V_l$. Here, $\Delta P = P_l - P_g$, is the difference between the pressure of the liquid and the gas. Given that the pressure of the gas in our simulations is, essentially, the vapor pressure of (SPC) water, $\Delta P \sim P_l$ and

$E = -P_l V_l + \sigma_{eff} A_{nom} + \sigma^{lg} A^{lg} + \text{const}$ (S8).

Here, we neglected the solid-gas interface terms as typically it is much smaller than the solid-liquid and liquid-gas terms. For the same reason, within our approximation, the length of the triple line is constant during the intrusion, and its contribution to the intrusion pressure can be neglected.

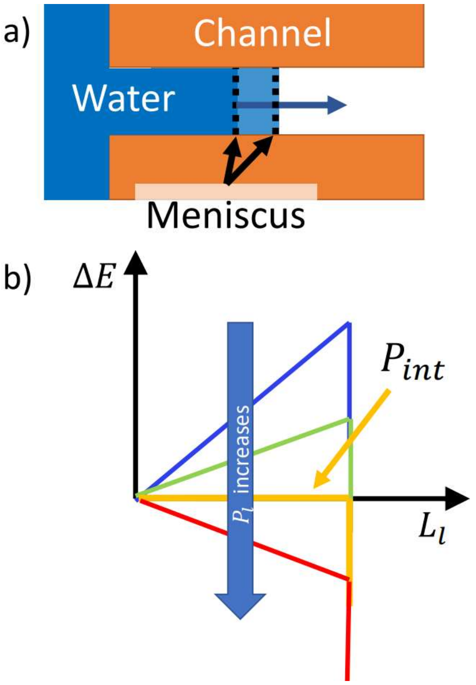

Figure S16. a) Cartoon of the continuum liquid at the basis of the capillary theory of intrusion. The liquid enters the cylindrical pore modelling ITT/mITT channels with an (almost) flat meniscus, which retains the same shape all along intrusion. b) Sketch of the variation of the grand potential (ΔE) with the position of the meniscus (L_l). ΔE depends linearly on L_l (top equation of Eq. S9) until the meniscus reaches the liquid entering from the opposite aperture of the cylinder (bottom equation of Eq. S9), when the grand potential suddenly drops because of the cancellation of the liquid-gas interface

In the following, we assume that the meniscus does not change shape along with intrusion, apart from joining with the other meniscus when it meets the liquid entering from the other end of the channel or reaches the channel end itself. We are aware that the assumption that the meniscus does not change shape during intrusion is, generally speaking, wrong, as the works of some of the authors of the present research have proven.[43,46,47] Nevertheless, i) to take into account the change of morphology of the meniscus inside 18MR channels, one should solve continuum equations of capillarity numerically, which would make our results certainly more accurate but more complex to analyze, thus undermining one of the objectives of this simple analysis. Moreover, the departure from the flat meniscus shape gives a sizable contribution to the grand potential only for very short channels, which is not the objective of this analysis.

Considering the formula of the area and the volume of a cylinder, $V_l = \pi r^2 L_l$ and $A_{nom} = 2\pi r L_l$, where r is the radius of the cylinder modelling 18MR channels and L_l is the depth of the liquid meniscus in the channel, Eq. S8 can be cast into:

$$E(L_l) = \left(-P_l \pi r^2 + \sigma_{eff} 2\pi r\right) L_l$$
$$+\sigma^{lg} A^{lg} + \text{const} \qquad \text{(S9)}.$$

From S8, within the hypothesis that the meniscus remains flat all along with intrusion, one can compute the variation of the grand potential $\Delta E(L_l) = E(L_l) - \mathrm{E}(0)$ with the position of the meniscus L_l, where E(0) is the grand potential of the system before intrusion (meniscus at $L_l = 0$ – see Figure S16):

$$\Delta E(L_l) = \begin{cases} \left(-P_l \pi r^2 + \sigma_{eff} 2\pi r\right) L_l \\ \left(-P_l \pi r^2 + \sigma_{eff} 2\pi r\right) \mathrm{L} - \sigma^{lg} A^{lg} \end{cases} \qquad \text{(S10)}.$$

The equations at the top and bottom of Eq. S10 are valid when the liquid does not and does completely intrude the channel, respectively. In the latter case, when at the end of the process one lacks the meniscus that merged with the liquid entering from the other aperture of the cylinder, the grand potential is diminished by a quantity corresponding to the liquid-gas interface energy, $\sigma^{lg} A^{lg}$.

Macroscopically, intrusion takes place when there is no barrier,[35,46] i.e., when $\Delta E(L_l) \leq 0$ for any $L_l > 0$, which gives us the condition for intrusion (top equation of Eq. S10)

$$\Delta E(L_l) = 0 = -\left(P_l \pi r^2 + \sigma_{eff} 2\pi r\right) L_l \qquad \text{(S11)},$$

From which one obtains

$$\sigma_{eff} = \frac{P_l r}{2} \qquad \text{(S12)}.$$

Thus, from the intrusion pressure measured in simulations and the radius of 18MR channels, one can estimate σ_{eff} for ITT and mITT: $\sigma_{eff}^{ITT} = 19.5$ mJ/m^2 and $\sigma_{eff}^{mITT} = 26$ mJ/m^2. From σ_{eff}^{ITT} and σ_{eff}^{mITT}, using Eq. S5, one obtains σ_{eff}^{mITT}=114° and $\sigma_{eff}^{ITT} =$ 108°, confirming that the shallow apertures of ITT reduce the hydrophobicity of the material by ~6° of the corresponding effective contact angle of the inner surface with respect to mITT.

To summarize, because lateral apertures are thin, they get wet during intrusion in the main pore. Remarkably, this, because of the bridging with water on the other side of the aperture, reduces hydrophobicity instead of increasing it, as typically happens with (nano)cavities and (nano)roughness.[39,40]

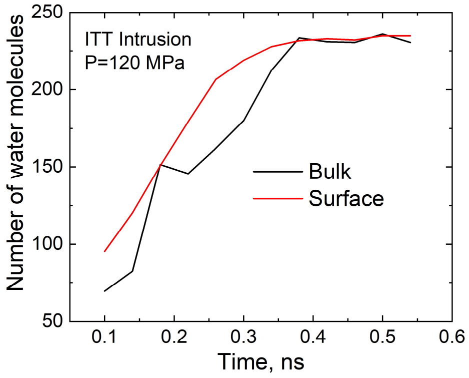

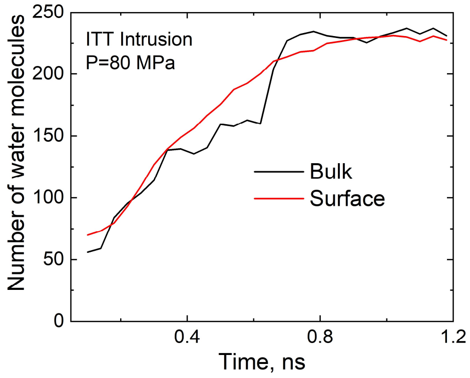

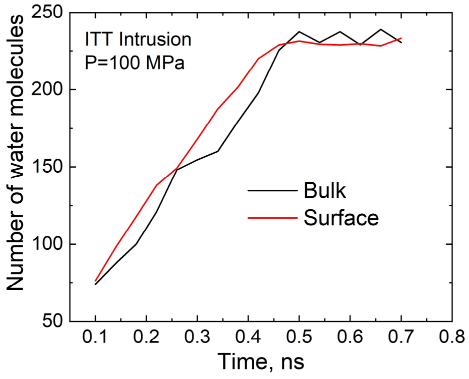

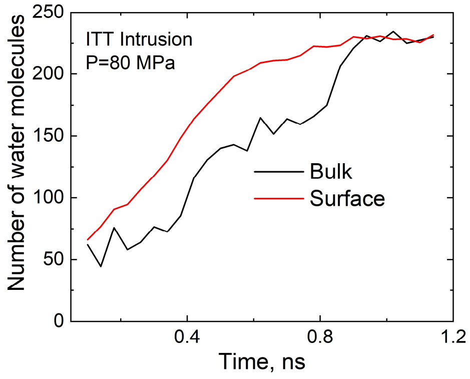

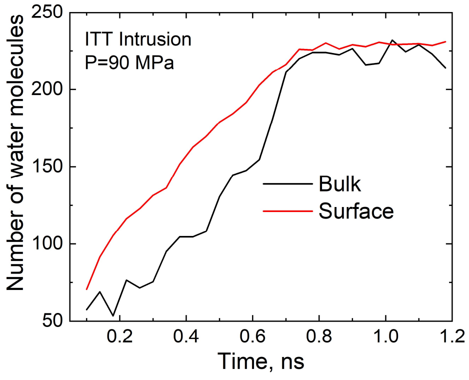

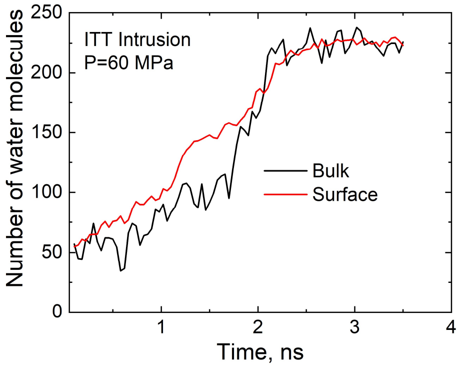

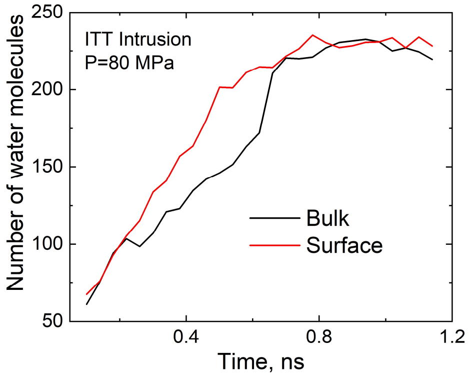

Figure S17. Kinetics of water intrusion in ITT-type zeolite.

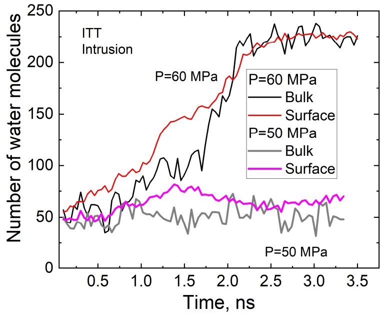

Figure S18. Kinetics of water intrusion in ITT-type zeolite at a pressure close to the intrusion pressure 50 and 60MPa.

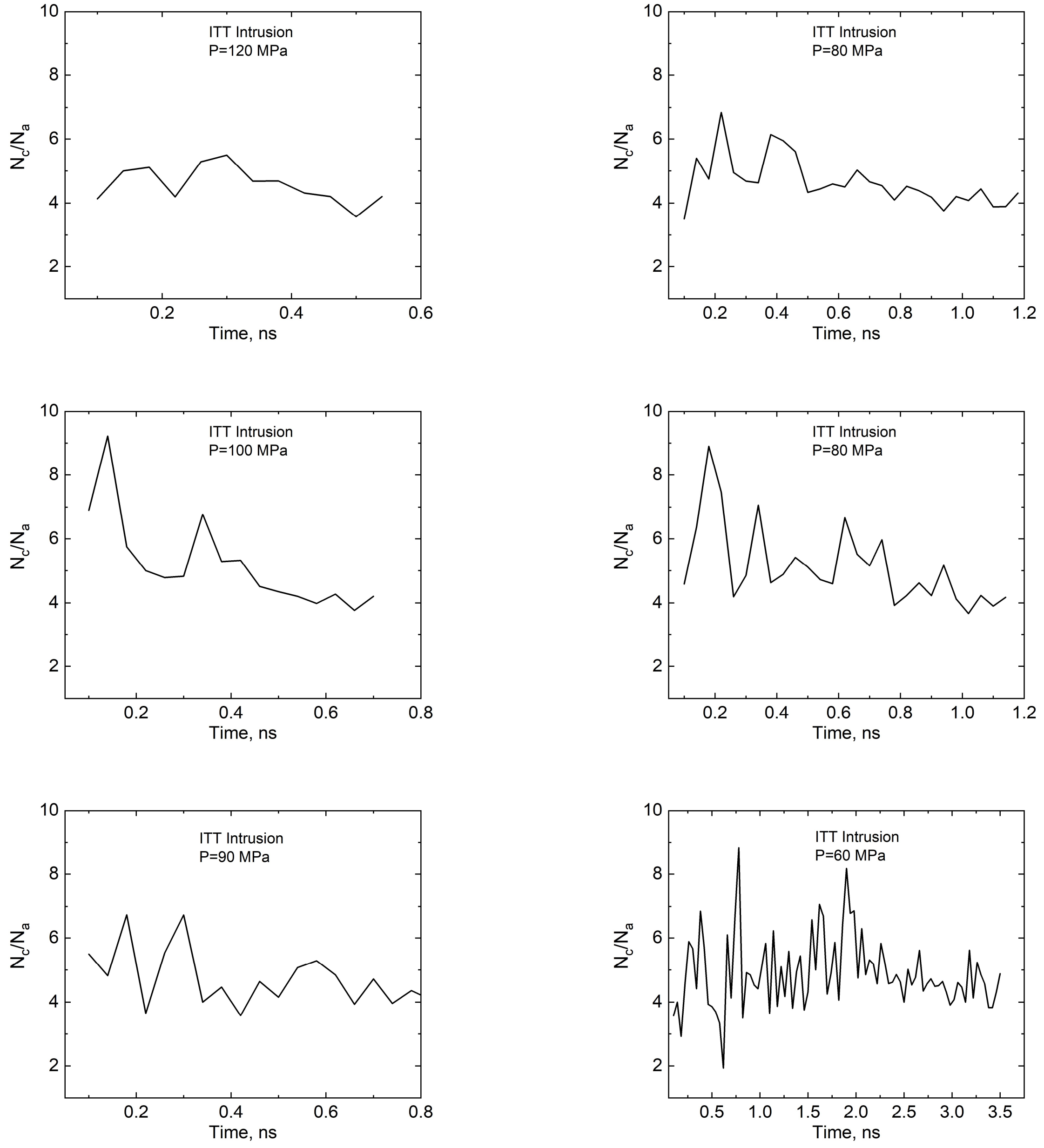

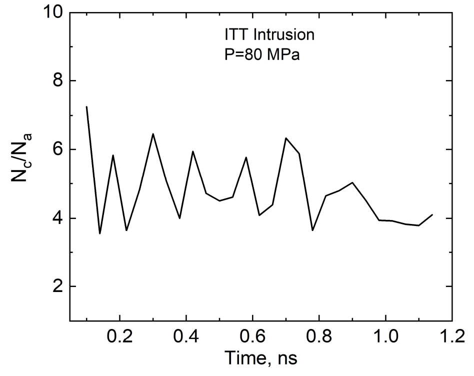

Figure S19. Kinetics of water intrusion in ITT-type zeolite. Changes of the proportion of molecules in the bulk channel to molecules in adjoined pores with intrusion time.

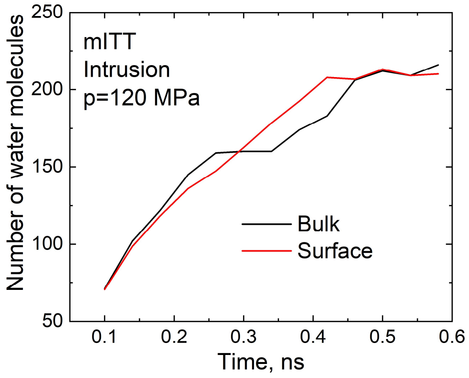

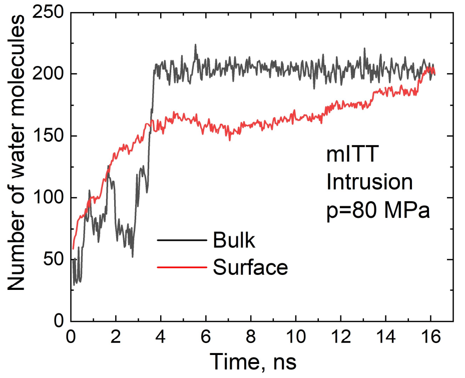

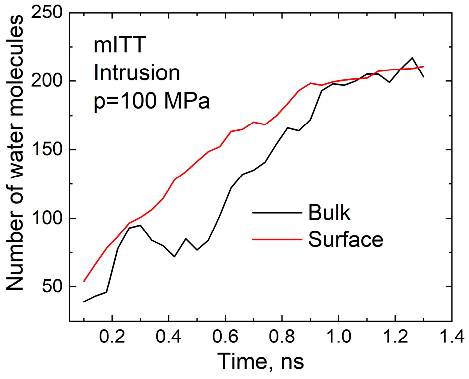

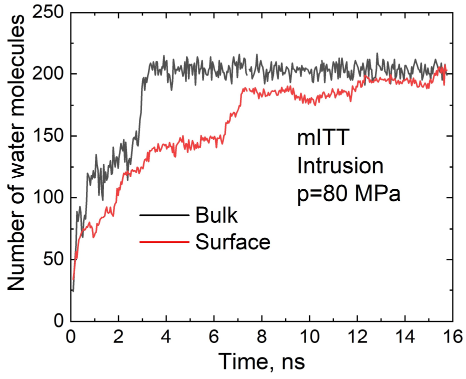

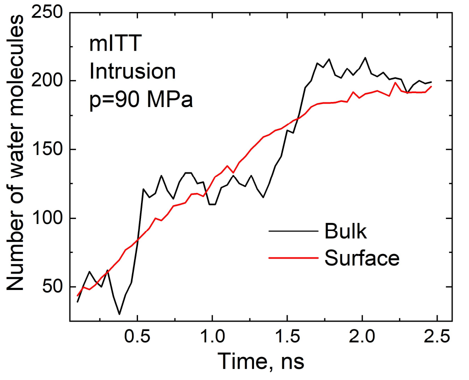

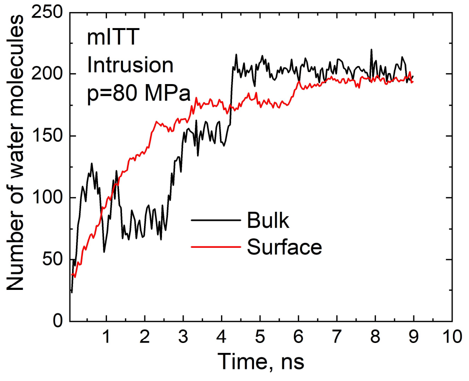

Figure S20. Kinetics of water intrusion in mITT-type zeolite. Simulations at 80 MPa, the lowest value simulated just above the intrusion pressure, the pressure condition that we used to analyze the intrusion mechanism, are repeated three times starting from different initial conditions (kinetic curves are shown here and one in the main text).

Movies S1 and S2 show intrusion trajectories of selected ITT 18 MR channels, together with relevant water molecules wetting adjacent 10 MR windows. The videos refer to trajectories at P = 60 MPa. The time of the simulation is ca. 2.5 ns, the time-step between frames is 40 ps. Intruding water is shown by the surface enveloping the liquid, using different colors for the liquid intruding different channels. Water molecules bridging two channels is shown by green spheres. Water molecules bridging surface channels and the water surrounding the crystallite, together with selection water of the surrounding water itself, is shown as orange spheres.

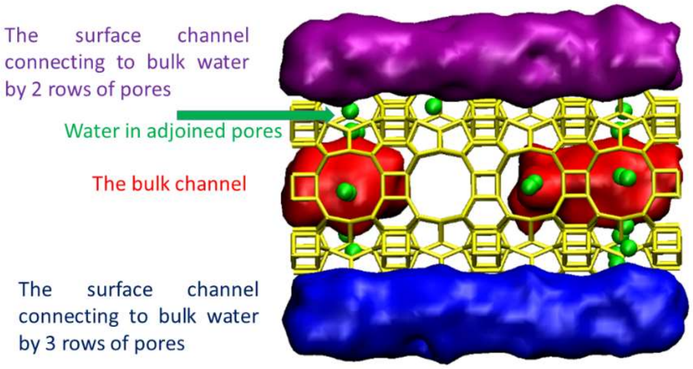

Figure S13. (Movie S1) lateral view during water intrusion in three neighboring channels. It is seen that lateral apertures allow bridging between the water inside the channels

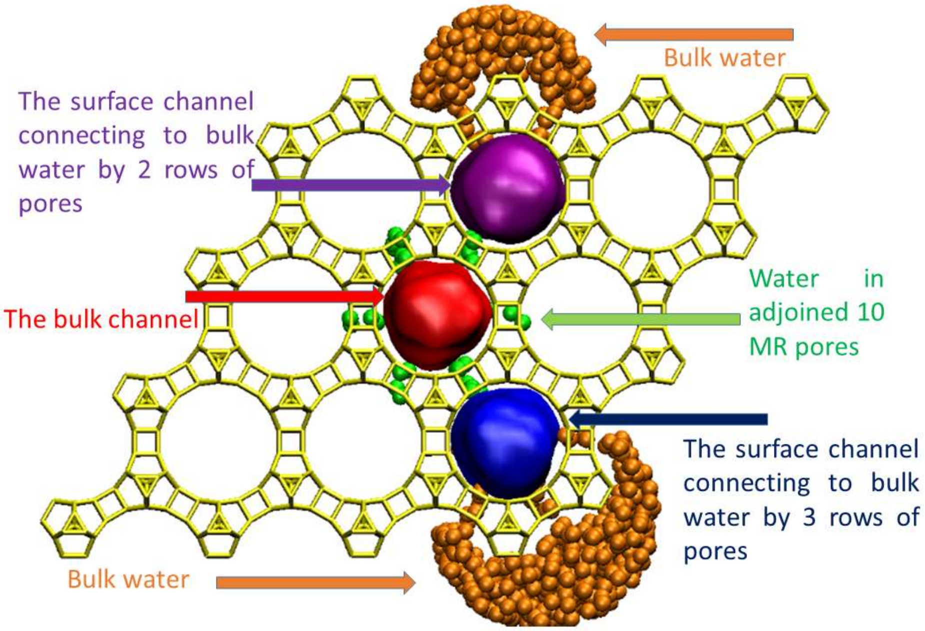

Figure S21. (Movie S2) top view during water intrusion in three neighboring channels. This view helps appreciate the bridging between surface channels and bulk water surrounding the crystallite.

Bibliography

(1) Bezus, A. G.; Kiselev, A. V.; Lopatkin, A. A.; Du, P. Q. Molecular Statistical Calculation of the Thermodynamic Adsorption Characteristics of Zeolites Using the Atom-Atom Approximation. Part 1. - Adsorption of Methane by Zeolite NaX. *J. Chem. Soc. Faraday Trans. 2 Mol. Chem. Phys.* **1978**, *74*, 367–379. https://doi.org/10.1039/F29787400367.

(2) Fuchs, A. H.; Cheetham, A. K. Adsorption of Guest Molecules in Zeolitic Materials: Computational Aspects. *J. Phys. Chem. B* **2001**, *105* (31), 7375–7383. https://doi.org/10.1021/jp010702q.

(3) Coudert, F. X.; Cailliez, F.; Vuilleumier, R.; Fuchs, A. H.; Boutin, A. Water Nanodroplets Confined in Zeolite Pores. *Faraday Discuss.* **2008**, *141*, 377–398. https://doi.org/10.1039/b804992k.

(4) Strzempek, M.; Tarach, K.; Góra-Marek, K.; Rey, F.; Palomino, M.; Valencia, S.; Piskorz, W. Multiscale Exploration of Hydrocarbon Adsorption and Hopping through ZSM-5 Channels - From Monte Carlo Modelling to Experiment. *Phys. Chem. Chem. Phys.* **2021**, *23* (4), 2981–2990. https://doi.org/10.1039/d0cp05128d.

(5) Bai, P.; Tsapatsis, M.; Siepmann, J. I. TraPPE-Zeo: Transferable Potentials for Phase Equilibria Force Field for All-Silica Zeolites. *J. Phys. Chem. C* **2013**, *117* (46), 24375–24387. https://doi.org/10.1021/jp4074224.

(6) Bushuev, Y. G.; Sastre, G.; Julia, J. V. De; Ga, J. Water – Hydrophobic Zeolite Systems. *J. Phys. Chem. C* **2012**, *116* (47), 24916–24929.

(7) Sastre, G.; Gale, J. D. Derivation of an Interatomic Potential for Fluoride-Containing Microporous Silicates and Germanates. *Chem. Mater.* **2005**, *17* (4), 730–740. https://doi.org/10.1021/cm048406o.

(8) Combariza, A. F.; Gomez, D. A.; Sastre, G. Simulating the Properties of Small Pore Silica Zeolites Using Interatomic Potentials. *Chem. Soc. Rev.* **2013**, *42* (1), 114–127. https://doi.org/10.1039/c2cs35243e.

(9) Sastre, G.; Corma, A. Rings and Strain in Pure Silica Zeolites. *J. Phys. Chem. B* **2006**, *110* (36), 17949–17959.

(10) Isaac, C.; Confalonieri, G.; Nouali, H.; Paillaud, J. L.; Arletti, R.; Daou, T. J.; Ryzhikov, A. Unusual High-Pressure Intrusion-Extrusion Behavior of Electrolyte Solutions in Mu-26, a Pure Silica Zeolite of Topology STF. *Microporous Mesoporous Mater.* **2020**, *298* (January), 110047. https://doi.org/10.1016/j.micromeso.2020.110047.

(11) Bushuev, Y. G.; Sastre, G. Atomistic Simulations of Water and Organic Templates Occluded during the Synthesis of Zeolites. *Microporous Mesoporous Mater.* **2010**, *129* (1–2), 42–53. https://doi.org/10.1016/j.micromeso.2009.08.031.

(12) Bushuev, Y. G.; Sastre, G. Feasibility of Pure Silica Zeolites. *J. Phys. Chem. C* **2010**, *114* (45), 19157–19168. https://doi.org/10.1021/jp107296e.

(13) Santoro, M.; Veremeienko, V.; Polisi, M.; Fantini, R.; Alabarse, F.; Arletti, R.; Quatieri, S.; Svitlyk, V.; Van Der Lee, A.; Rouquette, J.; Alonso, B.; Di Renzo, F.; Coasne, B.; Haines, J. Insertion and Confinement of H2O in Hydrophobic Siliceous Zeolites at High Pressure. *J. Phys. Chem. C* **2019**, *123* (28), 17432–17439. https://doi.org/10.1021/acs.jpcc.9b04860.

(14) Cygan, R. T.; Liang, J. J.; Kalinichev, A. G. Molecular Models of Hydroxide, Oxyhydroxide, and Clay Phases and the Development of a General Force Field. *J. Phys. Chem. B* **2004**, *108* (4), 1255–1266. https://doi.org/10.1021/jp0363287.

(15) Emami, F. S.; Puddu, V.; Berry, R. J.; Varshney, V.; Patwardhan, S. V.; Perry, C. C.; Heinz, H. Force Field and a Surface Model Database for Silica to Simulate Interfacial Properties in Atomic Resolution. *Chem. Mater.* **2016**, *28* (1), 406–407. https://doi.org/https://doi.org/10.1021/cm500365c.

(16) H. J. C. Berendsen; Postma, J. P. M.; Gunsteren, W. F. van; Hermans, and J. No Title. In *Intermolecular Forces*; Pullman, B., Ed.; Reidel: Dordrecht, 1982; p 331.

(17) Molinero, V.; Moore, E. B. Water Modeled as an Intermediate Element between Carbon and Silicon. *J. Phys. Chem. B* **2009**, *113* (13), 4008–4016. https://doi.org/10.1021/jp805227c.

(18) Guillemot, L.; Biben, T.; Galarneau, A.; Vigier, G.; Charlaix, É. Activated Drying in Hydrophobic Nanopores and the Line Tension of Water. *Proc. Natl. Acad. Sci. U. S. A.* **2012**, *109* (48), 19557–19562. https://doi.org/10.1073/pnas.1207658109.

(19) Melchionna, S.; Ciccotti, G.; Lee, B. Hoover NPT Dynamics for Systems Varying in Shape and Size. *Mol. Phys.* **1993**, *78* (3), 533–544.

(20) Wu, Y.; Tepper, H. L.; Voth, G. A. Flexible

Simple Point-Charge Water Model with Improved Liquid-State Properties. *J. Chem. Phys.* **2006**, *124* (2). https://doi.org/10.1063/1.2136877.

(21) Plimpton, S. Fast Parallel Algorithms for Short-Range Molecular Dynamics. *J. Comput. Phys.* 1995, pp 1–19. https://doi.org/10.1006/jcph.1995.1039.

(22) Thompson, A. P.; Aktulga, H. M.; Berger, R.; Bolintineanu, D. S.; Brown, W. M.; Crozier, P. S.; in 't Veld, P. J.; Kohlmeyer, A.; Moore, S. G.; Nguyen, T. D.; Shan, R.; Stevens, M. J.; Tranchida, J.; Trott, C.; Plimpton, S. J. LAMMPS - a Flexible Simulation Tool for Particle-Based Materials Modeling at the Atomic, Meso, and Continuum Scales. *Comput. Phys. Commun.* **2022**, *271*, 108171. https://doi.org/10.1016/j.cpc.2021.108171.

(23) Lu, J.; Jacobson, L. C.; Perez Sirkin, Y. A.; Molinero, V. High-Resolution Coarse-Grained Model of Hydrated Anion-Exchange Membranes That Accounts for Hydrophobic and Ionic Interactions through Short-Ranged Potentials. *J. Chem. Theory Comput.* **2017**, *13* (1), 245–264. https://doi.org/10.1021/acs.jctc.6b00874.

(24) Gyawali, G.; Sternfield, S.; Kumar, R.; Rick, S. W. Coarse-Grained Models of Aqueous and Pure Liquid Alkanes. *J. Chem. Theory Comput.* **2017**, *13* (8), 3846–3853. https://doi.org/10.1021/acs.jctc.7b00389.

(25) Smit, B.; Maesen, T. L. M. Molecular Simulations of Zeolites: Adsorption, Diffusion, and Shape Selectivity. *Chem. Rev.* **2008**, *108* (10), 4125–4184. https://doi.org/10.1021/cr8002642.

(26) Trzpit, M.; Soulard, M.; Patarin, J.; Desbiens, N.; Cailliez, F.; Boutin, A.; Demachy, I.; Fuchs, A. H. *Influence of Defects on the Water Intrusion in Silicalite-1 Zeolite: Confrontation of Experimental and Molecular Simulation Results*; Elsevier B.V., 2008; Vol. 174. https://doi.org/10.1016/S0167-2991(08)80262-5.

(27) Tzanis, L.; Trzpit, M.; Soulard, M.; Patarin, J. High Pressure Water Intrusion Investigation of Pure Silica 1D Channel AFI, MTW and TON-Type Zeolites. *Microporous Mesoporous Mater.* **2011**, *146* (1–3), 119–126. https://doi.org/10.1016/j.micromeso.2011.03.043.

(28) Trzpit, M.; Soulard, M.; Patarin, J.; Desbiens, N.; Cailliez, F.; Boutin, a; Demachy, I.; Fuchs, a H. The Effect of Local Defects on Water Adsorption in Silicalite-1 Zeolite: A Joint Experimental and Molecular Simulation Study. *Langmuir* **2007**, *23* (20), 10131–10139. https://doi.org/10.1021/la7011205.

(29) Zhou, T.; Bai, P.; Siepmann, J. I.; Clark, A. E. Deconstructing the Confinement Effect upon the Organization and Dynamics of Water in Hydrophobic Nanoporous Materials: Lessons Learned from Zeolites. *J. Phys. Chem. C* **2017**, *121* (40), 22015–22024. https://doi.org/10.1021/acs.jpcc.7b04991.

(30) Karbowiak, T.; Saada, M. A.; Rigolet, S.; Ballandras, A.; Weber, G.; Bezverkhyy, I.; Soulard, M.; Patarin, J.; Bellat, J. P. New Insights in the Formation of Silanol Defects in Silicalite-1 by Water Intrusion under High Pressure. *Phys. Chem. Chem. Phys.* **2010**, *12* (37), 11454–11466. https://doi.org/10.1039/c000931h.

(31) Confalonieri, G.; Daou, T. J.; Nouali, H.; Arletti, R.; Ryzhikov, A. Energetic Performance of Pure Silica Zeolites under High-Pressure Intrusion of LiCl Aqueous Solutions: An Overview. *Molecules* **2020**, *25* (9). https://doi.org/10.3390/molecules25092145.

(32) Lefevre, B.; Saugey, A.; Barrat, J. L.; Bocquet, L.; Charlaix, E.; Gobin, P. F.; Vigier, G. Intrusion and Extrusion of Water in Hydrophobic Mesopores. *J. Chem. Phys.* **2004**, *120* (10), 4927–4938. https://doi.org/10.1063/1.1643728.

(33) Giacomello, A.; Chinappi, M.; Meloni, S.; Casciola, C. M. Geometry as a Catalyst: How Vapor Cavities Nucleate from Defects. *Langmuir* **2013**, *29* (48), 14873–14884. https://doi.org/10.1021/la403733a.

(34) Bonella, S.; Meloni, S.; Ciccotti, G. Theory and Methods for Rare Events. *Eur. Phys. J. B* **2012**, *85* (3), 1–19. https://doi.org/10.1140/epjb/e2012-20366-2.

(35) Meloni, S.; Giacomello, A.; Casciola, C. M. Focus Article: Theoretical Aspects of Vapor/Gas Nucleation at Structured Surfaces. *J. Chem. Phys.* **2016**, *145* (21). https://doi.org/10.1063/1.4964395.

(36) Giacomello, A.; Casciola, C. M.; Grosu, Y.; Meloni, S. Liquid Intrusion in and Extrusion from Non-Wettable Nanopores for Technological Applications. *Eur. Phys. J. B* **2021**, *94* (8), 1–24. https://doi.org/10.1140/epjb/s10051-021-00170-3.

(37) Uribe-romo, F. J.; Knobler, C. B.; Keeffe, M. O.; Yaghi, O. M. Capture Properties of Zeolitic Imidazolate Frameworks. *Acc. Chem. Res.* **2010**, *43* (1), 58–67.

(38) Mortada, B.; Chaplais, G.; Veremeienko, V.; Nouali, H.; Marichal, C.; Patarin, J. Energetic Performances of ZIF-8 Derivatives: Impact of

the Substitution (Me, Cl, or Br) on Imidazolate Linker. *J. Phys. Chem. C* **2018**, *122* (7), 3846–3855. https://doi.org/10.1021/acs.jpcc.7b08999.

(39) Wenzel, R. N. RESISTANCE OF SOLID SURFACES TO WETTING BY WATER. *Ind. Eng. Chem.* **1936**, *28* (8), 988–994. https://doi.org/10.1021/ie50320a024.

(40) Cassie, A. B. D.; Baxter, S. Wettability of Porous Surfaces. *Trans. Faraday Soc.* **1944**, *40* (5), 546–551. https://doi.org/10.1039/tf9444000546.

(41) Vega, C.; De Miguel, E. Surface Tension of the Most Popular Models of Water by Using the Test-Area Simulation Method. *J. Chem. Phys.* **2007**, *126* (15). https://doi.org/10.1063/1.2715577.

(42) Sharma, S.; Debenedetti, P. G. Evaporation Rate of Water in Hydrophobic Confinement. *Proc. Natl. Acad. Sci. U. S. A.* **2012**, *109* (12), 4365–4370. https://doi.org/10.1073/pnas.1116167109.

(43) Tinti, A.; Giacomello, A.; Grosu, Y.; Casciola, C. M. Intrusion and Extrusion of Water in Hydrophobic Nanopores. *Proc. Natl. Acad. Sci. U. S. A.* **2017**, *114* (48), E10266–E10273. https://doi.org/10.1073/pnas.1714796114.

(44) Tolman, R. C. Consideration of the Gibbs Theory of Surface Tension. *J. Chem. Phys.* **1948**, *16* (8), 758–774. https://doi.org/10.1063/1.1746994.

(45) Tolman, R. C. The Effect of Droplet Size on Surface Tension. *J. Chem. Phys.* **1949**, *17* (3), 333–337. https://doi.org/10.1063/1.1747247.

(46) Giacomello, A.; Chinappi, M.; Meloni, S.; Casciola, C. M. Metastable Wetting on Superhydrophobic Surfaces: Continuum and Atomistic Views of the Cassie-Baxter-Wenzel Transition. *Phys. Rev. Lett.* **2012**, *109* (22), 1–4. https://doi.org/10.1103/PhysRevLett.109.226102.

(47) Giacomello, A.; Meloni, S.; Müller, M.; Casciola, C. M. Mechanism of the Cassie-Wenzel Transition via the Atomistic and Continuum String Methods. *J. Chem. Phys.* **2015**, *142* (10). https://doi.org/10.1063/1.4913839.
